# Mesenchymal adenomatous polyposis coli plays critical and diverse roles in regulating lung development

**DOI:** 10.1186/s12915-015-0153-1

**Published:** 2015-06-20

**Authors:** Yongfeng Luo, Elie El Agha, Gianluca Turcatel, Hui Chen, Joanne Chiu, David Warburton, Saverio Bellusci, Bang-Ping Qian, Douglas B. Menke, Wei Shi

**Affiliations:** Saban Research Institute, Children’s Hospital Los Angeles, Los Angeles, CA 90027 USA; Department of Surgery, Keck School of Medicine, University of Southern California, Los Angeles, CA 90027 USA; Excellence Cluster Cardio-Pulmonary System, Justus Liebig University Giessen, 35392 Giessen, Hessen Germany; Institute of Fundamental Medicine and Biology, Kazan (Volga Region) Federal University, 420008 Kazan, Russian Federation; Spine Surgery, The Affiliated Drum Tower Hospital of Nanjing University Medical School, Nanjing, 210008 China; Department of Genetics, University of Georgia, Athens, GA 30602 USA

**Keywords:** Adenomatous polyposis coli, Lung branching morphogenesis, Lung development, Pulmonary circulation, Lung mesenchyme, Lung vasculogenesis, Versican, Ctnnb1

## Abstract

**Background:**

Adenomatous polyposis coli (Apc) is a tumor suppressor that inhibits Wnt/Ctnnb1. Mutations of *Apc* will not only lead to familial adenomatous polyposis with associated epithelial lesions, but will also cause aggressive fibromatosis in mesenchymal cells. However, the roles of Apc in regulating mesenchymal cell biology and organogenesis during development are unknown.

**Results:**

We have specifically deleted the *Apc* gene in lung mesenchymal cells during early lung development in mice. Loss of Apc function resulted in immediate mesenchymal cell hyperproliferation through abnormal activation of Wnt/Ctnnb1, followed by a subsequent inhibition of cell proliferation due to cell cycle arrest at G0/G1, which was caused by a mechanism independent of Wnt/Ctnnb1. Meanwhile, abrogation of *Apc* also disrupted lung mesenchymal cell differentiation, including decreased airway and vascular smooth muscle cells, the presence of Sox9-positive mesenchymal cells in the peripheral lung, and excessive versican production. Moreover, lung epithelial branching morphogenesis was drastically inhibited due to disrupted Bmp4-Fgf10 morphogen production and regulation in surrounding lung mesenchyme. Lastly, lung mesenchyme-specific *Apc* conditional knockout also resulted in altered lung vasculogenesis and disrupted pulmonary vascular continuity through a paracrine mechanism, leading to massive pulmonary hemorrhage and lethality at mid-gestation when the pulmonary circulation should have started.

**Conclusions:**

Our study suggests that Apc in lung mesenchyme plays central roles in coordinating the proper development of several quite different cellular compartments including lung epithelial branching and pulmonary vascular circulation during lung organogenesis.

**Electronic supplementary material:**

The online version of this article (doi:10.1186/s12915-015-0153-1) contains supplementary material, which is available to authorized users.

## Background

Lung development is a complex process, controlled by reciprocal interactions between mesenchymal and epithelial cells [1]. In mice, the primary lung epithelial buds undergo reiterated elongation and division from E10.5, a process called branching morphogenesis, to form a tree-like airway structure with coordinated differentiation of epithelial and mesenchymal cells along proximal-distal airways. Simultaneously, pulmonary vascular networks are formed by angiogenesis and vasculogenesis, and are eventually connected to the heart to establish the pulmonary circulation around E14 [2]. During lung branching morphogenesis, mesenchymal progenitor cells undergo active proliferation and differentiation, giving rise to diverse cell lineages, including airway and vascular smooth muscle cells, pericytes, and stromal fibroblasts. These mesenchyme-derived cell lineages not only provide a structural support for the formation of the branched airways and the vascular networks, but may also regulate the growth of epithelial and endothelial cells by generating various morphogenic signals such as bone morphogenetic proteins (Bmps), fibroblast growth factors (Fgfs), and Wnts [1]. However, the molecular and cellular mechanisms by which mesenchymal cells regulate early lung development are as yet incompletely understood.

Adenomatous polyposis coli (*Apc*) was originally discovered as a tumor suppressor gene, and loss of function mutation of *Apc* results in colon cancer [3]. Apc is a large protein containing multi-domains that interact with a variety of proteins, including Ctnnb1 (or β-catenin)/Axin in canonical Wnt signaling and microtubules [4]. Therefore, Apc plays a critical role in regulating many cellular processes, such as cell proliferation, differentiation, migration, and chromosomal segregation. Germline mutations of *Apc* will not only lead to familial adenomatous polyposis (FAP) with associated epithelial lesions, but will also cause aggressive fibromatosis (also called desmoid tumors) in mesenchymal cells [5]. However, the lower incidence and benign features of desmoid tumors in patients with *Apc* germline mutation suggest that Apc may regulate mesenchymal cell biology through a mechanism different from that in epithelial cells.

Homozygous mutation of *Apc* in mice leads to early embryonic lethality, and conditional knockout (CKO) of *Apc* in a variety of cell compartments other than mesenchyme suggests that Apc plays important roles in development of brain cortex, skin, and thymus [6, 7]. Abrogation of *Apc* in lung epithelial cells was found to disrupt differentiation of airway club cells and ciliated cells by upregulating the Wnt/Ctnnb1 pathway [8], while direct activation of Wnt/Ctnnb1 in mouse embryonic lung epithelia induces cell lineage switching to intestinal cell types [9]. Although numerous studies have focused on Apc in ectoderm and endoderm derived cells, expression of *Apc* in early embryonic lung mesenchyme was not detected [10], and therefore, the roles of Apc in developing lung mesenchymal cells have never been explored. Herein, we have specifically deleted the *Apc* gene in lung mesenchymal cells during mouse lung branching morphogenesis, and found that loss of Apc function resulted in more severe and earlier phenotypes than those seen in the lung epithelial *Apc* knockout, which include arrest of lung epithelial branching morphogenesis with condensed mesenchyme. An early rapid increase, followed by a decrease, in cell proliferation was observed in mesenchymal *Apc* CKO lung, due to Wnt/Ctnnb1-dependent and Wnt/Ctnnb1-independent mechanisms, respectively. Mesenchymal cell differentiation was also disturbed in the *Apc* CKO lung, such as reduced airway and vascular smooth muscle cell generation and the presence of Sox9-positive mesenchymal cell population in distal lung, as well as increased proteoglycan versican (Vcan) production. Interestingly, abnormality in both epithelial branching and endothelial network formation was also observed, which correlated with deregulation of growth factor production in mesenchymal cells (Bmp4, Fgf10, Igf1, and Angpt1). Eventually, failure to establish an intact pulmonary circulation in the *Apc* CKO mice led to massive lung hemorrhage and fetal lethality at mid-gestation. Therefore, our study suggests that Apc in lung mesenchyme plays central roles in coordinating the proper development of several quite different cellular compartments during lung organogenesis.

## Results

### Homozygous deletion, but not heterozygous deletion, of *Apc* resulted in ectopic activation of Wnt/Ctnnb1 in embryonic lung mesenchyme

**Fig. 1:**
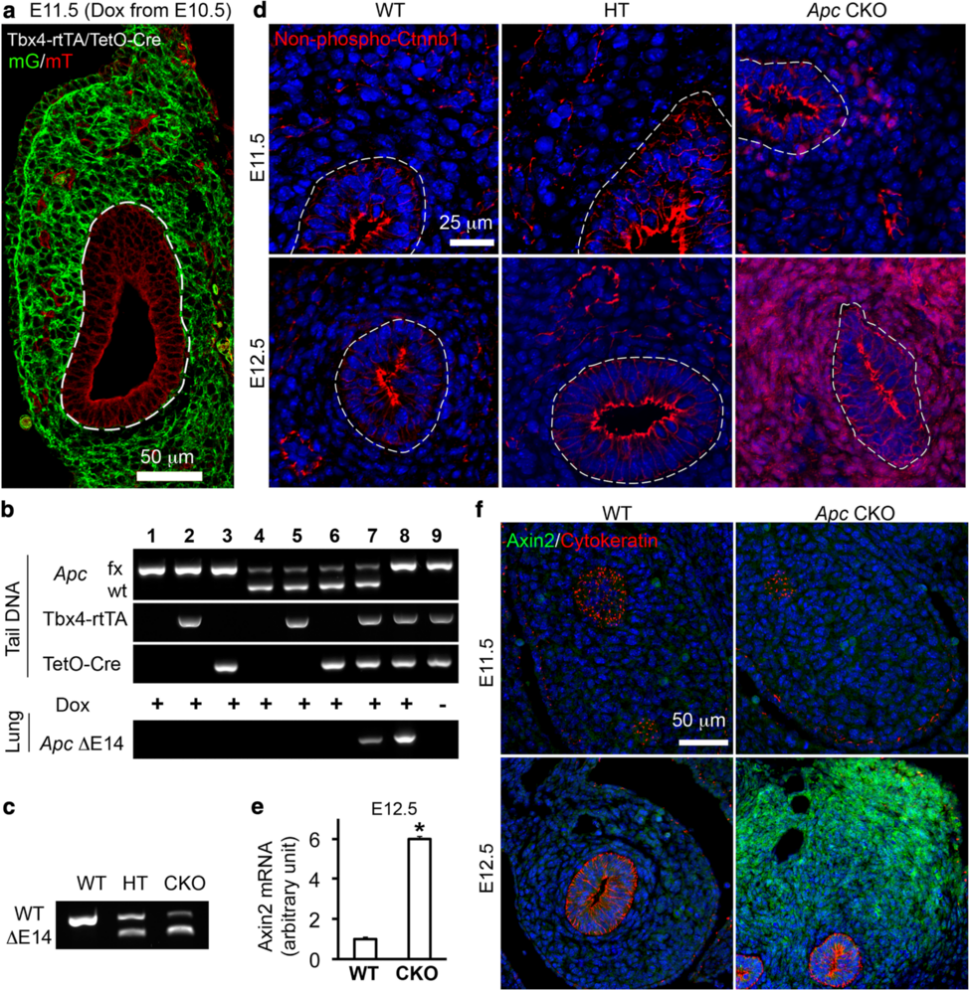
Embryonic lung mesenchymal deletion of *Apc* induced ectopic activation of Wnt/Ctnnb1 signaling. **a** Induced Cre expression specifically in embryonic lung mesenchyme by a *Tbx4* lung enhancer-driven Tet-On system was verified in an mT-mG reporter mouse lung (Tbx4-rtTA*/*TetO-Cre*/*mT-mG) one day after Dox administration (E10.5-E11.5). mG (green) was only expressed when upstream *floxed-mT* (red) was deleted by the induced Cre. **b** Lung specificity of *Apc* gene knockout at E11.5. Fetuses with different tail DNA genotypes were listed, and the heterozygous (lane 7) and homozygous (lane 8) deletions of floxed (fx)-exon 14 (ΔE14) were confirmed in lung tissue genomic DNAs. Leakage of *Apc* gene deletion in fetal lung was not detected in the absence of Dox induction (lane 9). **c** Truncation of *Apc* mRNA transcript in *Apc* conditional knockout lungs was verified by RT-PCR at E11.5. **d** Ectopic activation of Wnt/Ctnnb1 signaling in embryonic lung mesenchyme was detected by accumulated non-phospho (active) Ctnnb1 in both cytoplasm and nucleus of mesenchymal cells. Epithelial airway is highlighted with dashed line. **e-f** Increased Wnt canonical signaling activity in lung tissue was also verified by elevated *Axin2* expression using real-time PCR (**e**; *n* = 5 in each group, *P < 0.05) and immunofluorescence staining (**f**)

Using a *Tbx4* lung enhancer-driven Tet-On transgenic system generated in our lab [11], we were able to induce Cre expression specifically in mouse embryonic lung mesenchymal cells (Fig. 1a). The *Apc* CKO mice were induced during lung branching morphogenesis by administering doxycycline (Dox) from E10.5. Deletion of *Apc* exon 14 in lung tissue was verified at both genomic DNA and mRNA levels (Fig. 1b,1c). Since Apc is a negative regulator for the Wnt/Ctnnb1 canonical pathway [4], loss of Apc function is expected to result in abnormal activation of Ctnnb1. In our homozygous *Apc* CKO embryos, hyperactivation of Ctnnb1 was detected in embryonic lung mesenchyme, reflected by accumulation of non-phospho (Ser37/Thr41, also called active) Ctnnb1 at as early as E11.5 and significantly increased expression of *Axin2* (a Ctnnb1 downstream target gene) from E12.5 (Fig. 1d–1f and Additional file 1). In contrast, staining of Ctnnb1 in airway epithelial cells, mainly localized on apical cell membranes, was comparable between *Apc* CKO and WT lungs (Fig. 1d and Additional file 1), confirming mesenchymal specificity of altered Wnt signaling activity due to loss of Apc function. Interestingly, like the wild-type (WT) controls, heterozygous *Apc* CKO (HT) mice did not display detectable Wnt/Ctnnb1 activation in embryonic lung mesenchyme (Fig. 1d), suggesting that a single allele of WT *Apc* gene is sufficient to suppress abnormal activation of Wnt/Ctnnb1 signaling in these cells.

### Lung mesenchyme-specific *Apc* conditional knockout resulted in abnormal lung morphogenesis and fetal lethality at mid-gestation

**Fig. 2:**
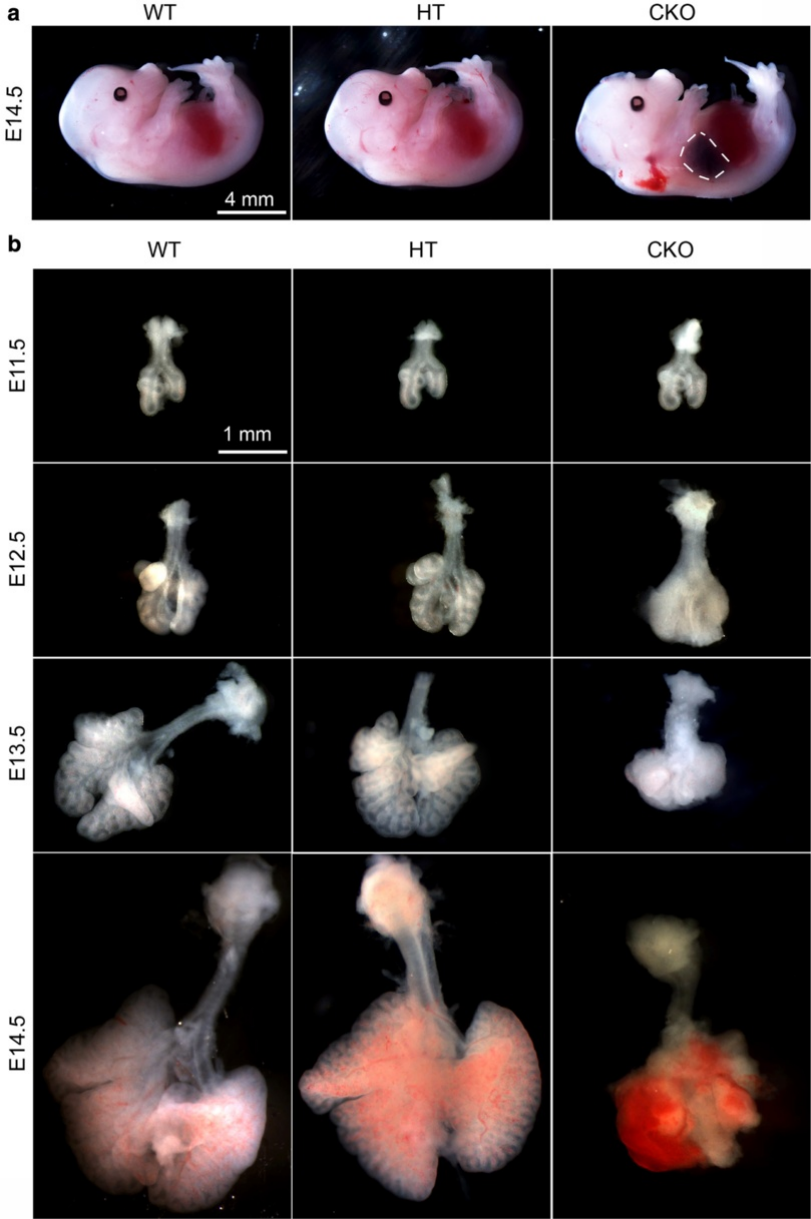
Mesenchymal-specific deletion of *Apc* resulted in lung malformation. **a** *Apc* CKO induced from E10.5 led to thoracic hemorrhage indicated by dashed line at E14.5. **b** Gross view of isolated lungs showed that the *Apc* CKO caused mesenchymal hyperplasia at E12.5, growth arrest at E13.5, and massive hemorrhage at E14.5. No significant change was observed between WT and *Apc* heterozygous conditional knockout (HT) lungs

Abrogation of *Apc* in lung mesenchyme starting from E10.5 did not affect the overall growth of the embryos/fetuses by comparison of their body sizes among different genotypes (Fig. 2a). However, the *Apc* CKO fetuses had severe chest hemorrhages with dark blue coloration by gross view at E14.5 (Fig. 2a), and died soon after E15.5. In order to determine the dynamic changes of the phenotypes, lungs of *Apc* CKO embryos were isolated from E11.5 to E14.5. As shown in Fig. 2b, most of the E11.5 *Apc* CKO lungs had normal epithelial domain branches surrounded with appropriate mesenchyme comparable to WT littermate controls. However, one day later (E12.5), airway branches of the *Apc* CKO lungs became difficult to see under a dissecting microscope due to a thickened and condensed mesenchymal compartment. The *Apc* CKO lung growth seemed fully arrested by E13.5, with a size and appearance similar to *Apc* CKO lungs at E12.5. In contrast, WT lungs grew rapidly with many airway branches. At E14.5, lung tissue destruction with massive hemorrhage was detected in *Apc* CKO mice, explaining the lung contusion observed within the whole body view (Fig. 2a). Interestingly, heterozygous *Apc* CKO did not result in any of the defects as described above, suggesting that one allele of *Apc* in lung mesenchymal cells is sufficient for proper early lung development, which is consistent with the unaltered Ctnnb1 activation shown above.

**Fig. 3:**
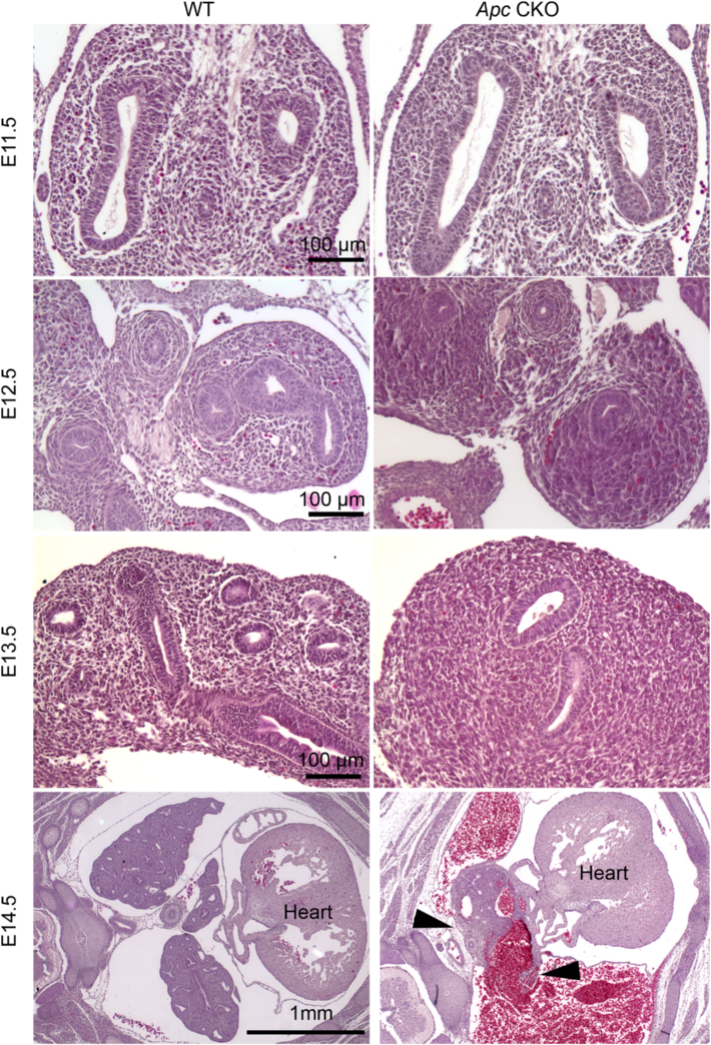
Dynamic changes of abnormal lung structure in *Apc* CKO lungs. There was no significant histological difference in E11.5 lungs between WT and *Apc* CKO mice. Aggregative growth of mesenchymal cells in *Apc* CKO lungs was apparent from E12.5. Lung tissue (arrowheads) was interspersed with massive areas of hemorrhage at E14.5

The abnormal histology of the *Apc* CKO lung was further analyzed in H&E-stained tissue sections (Fig. 3). Although no significant difference was observed between E11.5 WT and *Apc* CKO lungs, alteration of the mesenchymal structure in *Apc* CKO lungs was obvious starting from E12.5, with circumferentially orientated and condensed mesenchymal cells around epithelial tubes (see Additional file 2 for high magnification). There were fewer airway epithelial tubes in *Apc* CKO lungs starting from E12.5 (Fig. 3). At E14.5, a massive hemorrhage with peripheral lung tissue destruction was observed in *Apc* CKO fetuses (Fig. 3 and Additional file 3).

**Fig. 4:**
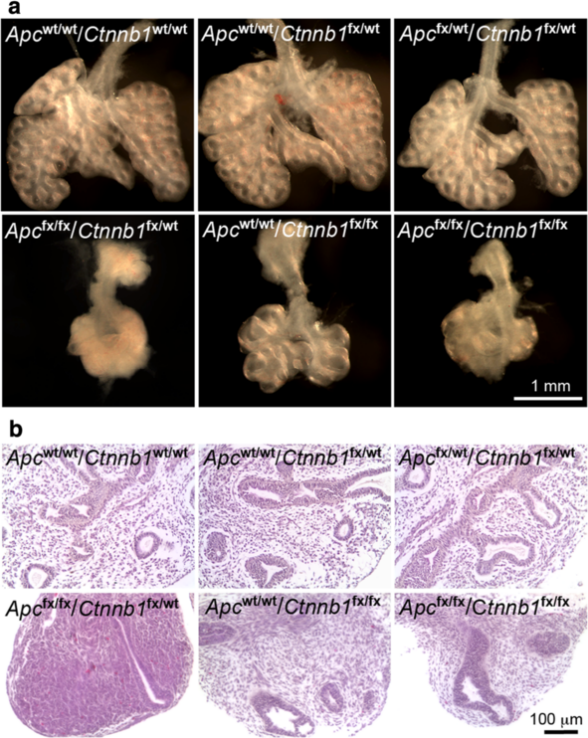
Phenotypic changes of E13.5 lungs with *Apc* and *Ctnnb1* double conditional knockout in lung mesenchyme. Lung tissues with indicated *Apc* and *Ctnnb1* genotypes were shown by gross view (**a**) and H&E-stained tissue sections (**b**). All fetal lungs shown here were positive for Tbx4-rtTA and TetO-Cre transgenes. Dox induction was started from E10.5

As mentioned above, Apc is a major negative regulator for Wnt/Ctnnb1 signaling, and the abnormal lung development in *Apc* CKO fetuses could be mediated by increased Ctnnb1 activation, which was indeed detected in E12.5 *Apc* CKO lung mesenchyme (Fig. 1). To further determine the potential molecular mechanism, we performed a rescue experiment by generating *Apc* CKO in combination with heterozygous or homozygous *Ctnnb1* knockout in developing lung mesenchyme. Surprisingly, reduction of *Ctnnb1* gene dosage by single allele deletion could not rescue lung phenotypes in *Apc* CKO fetuses (*Apc*^fx/fx^/*Ctnnb1*^fx/wt^/Tbx4-rtTA/TetO-Cre in Fig. 4), while null deletion of *Ctnnb1* alone (*Apc*^wt/wt^/*Ctnnb1*^fx/fx^/Tbx4-rtTA/TetO-Cre) resulted in reduced branching morphogenesis without change in mesenchymal cell density. Furthermore, null mutation of *Ctnnb1* in combination with *Apc* CKO (*Apc*^fx/fx^/*Ctnnb1*^fx/fx^/Tbx4-rtTA/TetO-Cre) resulted in complicated lung growth arrest, but no mesenchymal condensation (Fig. 4), suggesting that abnormal mesenchymal condensation of early *Apc* CKO fetal lung may be mediated by hyperactivation of Ctnnb1 following *Apc* deletion.

### Loss of Apc function in lung mesenchymal cells results in dynamic changes in cell proliferation that eventually lead to arrest of lung growth

**Fig. 5:**
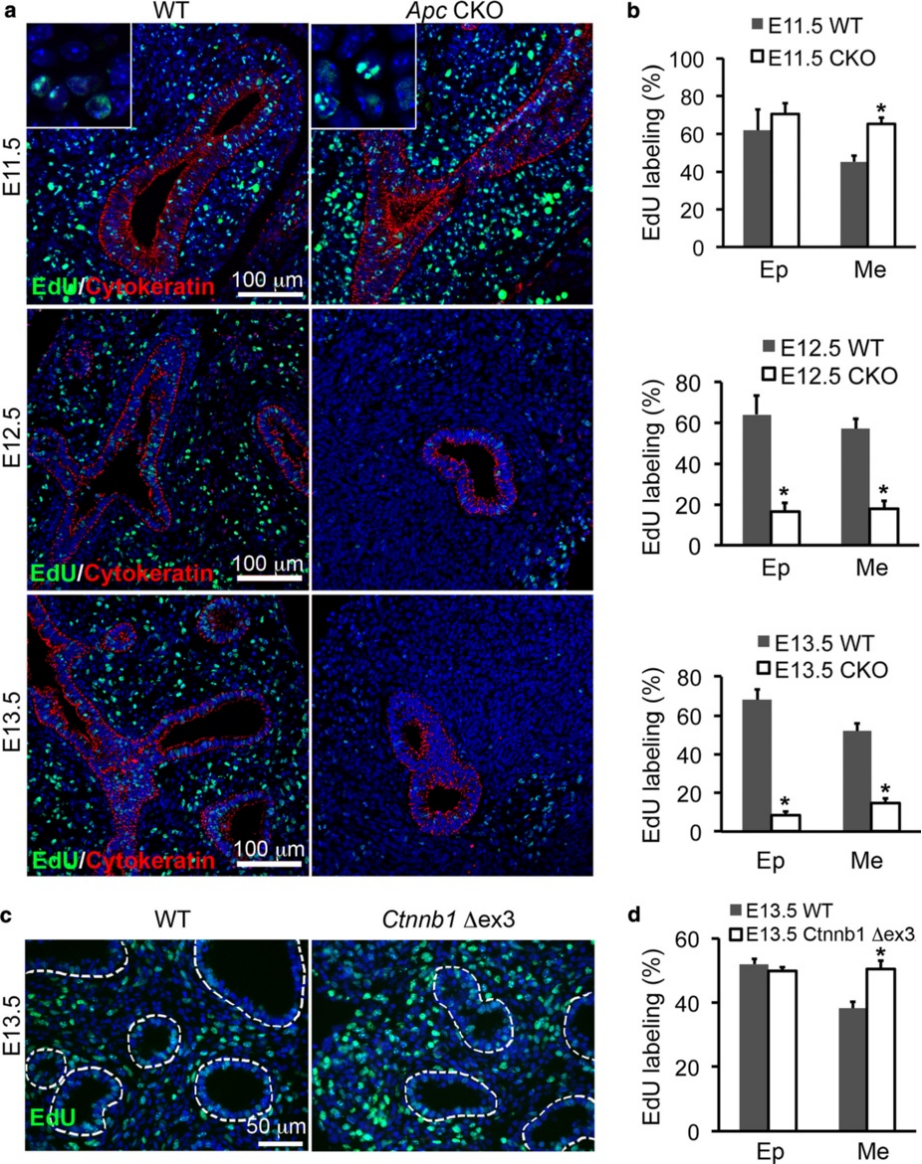
Dynamic changes in cell proliferation of *Apc* CKO lung. **a**-**b** Increased DNA synthesis, measured by EdU incorporation, in mesenchymal cells was found in lungs of *Apc* CKO mice at E11.5 (**a**-**b**), while decreased EdU labeling was observed in both epithelial and mesenchymal cells in *Apc* CKO lungs after E12.5. **c**-**d** Increased EdU incorporation persisted to E13.5 in lung mesenchyme when constitutively active *Ctnnb1* (or *Ctnnb1* Δex3) was expressed in mice with genotypes of *Tbx4-Cre* ^ERT^/*Ctnnb1* ^fx-ex3/+^ plus tamoxifen induction at E10.5. **a**, **c**: Tissue immunofluorescence staining; **b**, **d**: quantitative analyses of the immunostained cells. Ep: epithelial cells, Me: mesenchymal cells. *P < 0.05, *n* = 4

Given that Apc is a negative regulator for Wnt/Ctnnb1 signaling, loss of Apc function could have an important impact on mesenchymal cell proliferation. Short-term 5-ethynyl-2-deoxyuridine (EdU) incorporation was used to identify cells with active DNA synthesis. Consistently with activation of Wnt/Ctnnb1 signaling (Fig. 1), increased 2-hour EdU labeling was detected in lung mesenchymal cells rather than epithelial cells at E11.5, one day after *Apc* knockout induction (Fig. 5a,5b). However, while both epithelial and mesenchymal cells in WT embryonic lungs retained a steady rate of cell proliferation shown by 2-hour EdU labeling at E12.5 and E13.5, cell proliferation in both epithelial and mesenchymal cells of *Apc* CKO lungs was then significantly reduced (Fig. 5a,5b). Interestingly, mouse embryos with a lung mesenchyme-specific activation of Ctnnb1 from E10.5 (*Tbx4-Cre*^ERT2^/*Cnntb1*^floxed-ex3^ or *Ctnnb1* Δex3/+) had a sustained increase of lung mesenchymal cell proliferation, as detected by 2-hour EdU incorporation during early lung development (Fig. 5c,5d and Additional file 4), suggesting that subsequent reduction of cell proliferation in *Apc* CKO lungs after E12.5 is likely independent of aberrant Ctnnb1 activation.

**Fig. 6:**
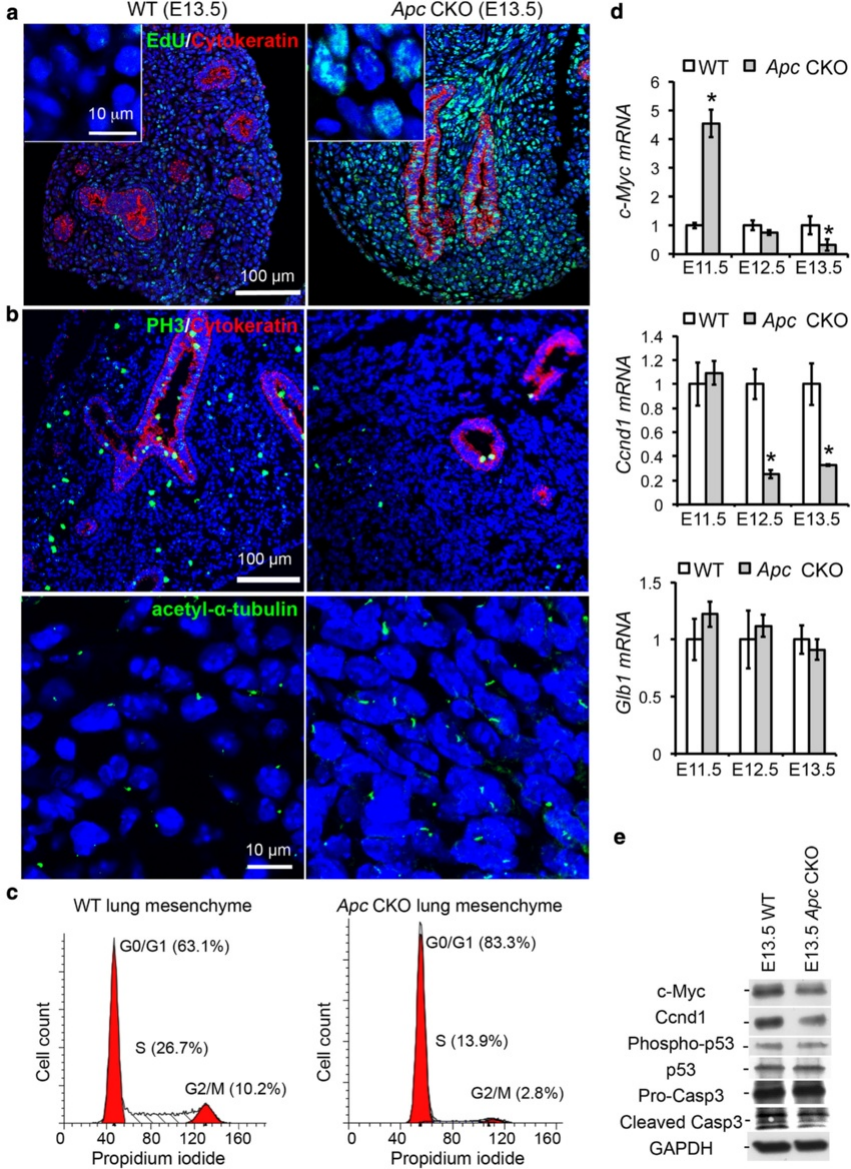
Cell cycle arrest at G0/G1 phase in E13.5 *Apc* CKO lung. **a** Significant reduction of cell division in E13.5 *Apc* CKO lung, indicated by increased cells that retain EdU DNA labeling at E11.5. Increased nuclear size (insert) was apparent in *Apc* CKO lung. **b** Immunostaining for PH3 and acetyl-α-tubulin, markers for metaphase and G0/G1 phase, respectively. **c** FACS analysis of DNA contents for the cells isolated from E13.5 *Apc* CKO or WT control lungs. **d** Expression of key genes involved in regulation of cell cycle and senescence, detected by real-time PCR. *P < 0.05, *n* = 4. **e** Protein levels of the related genes were detected by western blot

To further understand the related mechanisms, we performed a pulse-chase experiment with EdU labeling at E11.5 and detection of the labeled DNA at E13.5. In WT lung, only a few cells were still positive for early EdU labeling due to continuous cell division and rounds of DNA synthesis (Fig. 6a). In contrast, the majority of lung mesenchymal cells in *Apc* CKO lungs were still EdU-positive. Interestingly, the sizes of mesenchymal cell nuclei in E13.5 *Apc* CKO lungs (30.0 ± 3.7 μm^2^) were significantly larger than those of WT littermate controls (23.8 ± 2.7 μm^2^, *P* < 0.05) and those of E11.5 *Apc* CKO lungs (23.0 ± 3.2 μm^2^, *P* < 0.05). These data suggested that the cells with *Apc* deletion were subsequently arrested in the cell cycle. Since interaction between Apc and the plus-end of microtubules is reported to be essential for spindle formation and chromatin segregation at metaphase in cultured cells [4], cell cycle analysis of *Apc* knockout lung mesenchyme was then performed in order to provide a potential mechanism for altered cell proliferation. Surprisingly, most *Apc* CKO lung mesenchymal cells were negative for phosphorylated Histone 3 (PH3), a marker of metaphase. Instead, from E12.5, they had more primary cilia as detected by acetylated α-tubulin staining (Fig. 6b and Additional file 5A), which is a post-mitotic cellular structure existing in G0/G1 phase [12, 13]. These findings indicate that lung mesenchymal cells with *Apc* deletion are arrested at G0/G1 phase rather than metaphase. To further verify this, single cell suspensions prepared from E13.5 lung were assessed for their DNA contents using propidium iodide staining and fluorescence-activated cell sorting (FACS) analysis (Fig. 6c). 83 % of *Apc* CKO lung mesenchymal cells were in G0/G1 phase, a significantly higher percentage than that in the WT controls (63 %), while the cells at S and G2/M phases were relatively lower in *Apc* CKO lung (13.9 % and 2.8 %) than those (26.7 % and 10.2 %) in the WT controls. Therefore, the significant reduction of cell proliferation seen in the *Apc* CKO lung after E12.5 was not due to metaphase arrest, but rather due to more cells exiting the cell cycle.

Additional studies were performed to determine the cellular mechanisms underlying lung growth arrest, including cell senescence and apoptosis. The dynamic expression of key genes involved in the cell cycle was measured using real-time PCR (Fig. 6d). Interestingly, *c-Myc* expression was increased at E11.5, not changed at E12.5, and decreased at E13.5. The increased c-Myc protein expression in E11.5 *Apc* CKO fetal lung mesenchymal cells was also verified by immunostaining (Additional file 5B). Moreover, the mRNA level of *Ccnd1* (encoding cyclin D1) was significantly reduced from E12.5 to E13.5 in *Apc* CKO lungs. The changes of gene expression at E13.5 were further verified at the protein level by western blot (Fig. 6e). However, expression or activation of genes associated with cell senescence, *Glb1* (encoding SA-β-galactosidase) and phospho-p53 [14, 15], were not altered (Fig. 6d,e). In addition, activation of the apoptotic pathway and number of apoptotic cells, evaluated by caspase 3 activation and nuclear DNA fragmentation (Fig. 6e and Additional file 6), were not changed between *Apc* CKO lung and WT controls. Therefore, with apoptosis and senescence ruled out, the impaired growth of embryonic lung in *Apc* CKO fetuses is likely due to G0/G1 cell cycle arrest.

### Apc is essential for appropriate mesenchymal cell differentiation and some extracellular matrix production

**Fig. 7:**
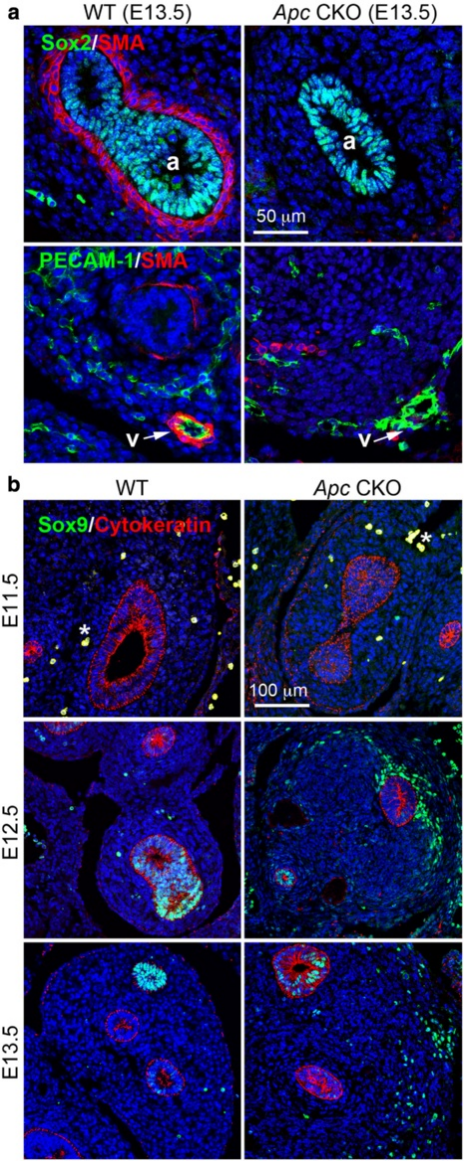
Abrogation of *Apc* altered lung mesenchymal cell differentiation. **a** Deficient smooth muscle cell differentiation, detected by SMA immunostaining, was observed in both proximal airways (a) and vasculature (v) of E13.5 *Apc* CKO lung, which were marked by positively stained Sox2 and PECAM1, respectively. **b** Abnormal Sox9 expression was seen in the mesenchymal cells disseminated in peripheral lung of *Apc* CKO after E12.5. Lung epithelial cells were marked by cytokeratin staining. Cell nuclei were counterstained with DAPI (blue). *Hematopoietic cells with non-specific autofluorescence in E11.5 lungs

During branching morphogenesis, mesenchymal cells surrounding proximal airways or vasculature, marked by epithelial Sox2 or endothelial PECAM1, differentiate into airway or vascular smooth muscle cells. However, abrogation of *Apc* significantly inhibited both proximal airway and vasculature smooth muscle cell differentiation, as detected by α-smooth muscle actin (SMA) staining in Fig. 7a. Interestingly, some mesenchymal cells in E13.5 peripheral lung of *Apc* CKO fetuses expressed Sox9, while only peripheral airway epithelial cells in the WT lung were positive for Sox9 staining at this stage (Fig. 7b). Although the exact identity of these Sox9-positive mesenchymal cells is unknown, commitment and differentiation of lung mesenchymal cell lineages in *Apc* CKO lung appear to be severely disrupted.

**Fig. 8:**
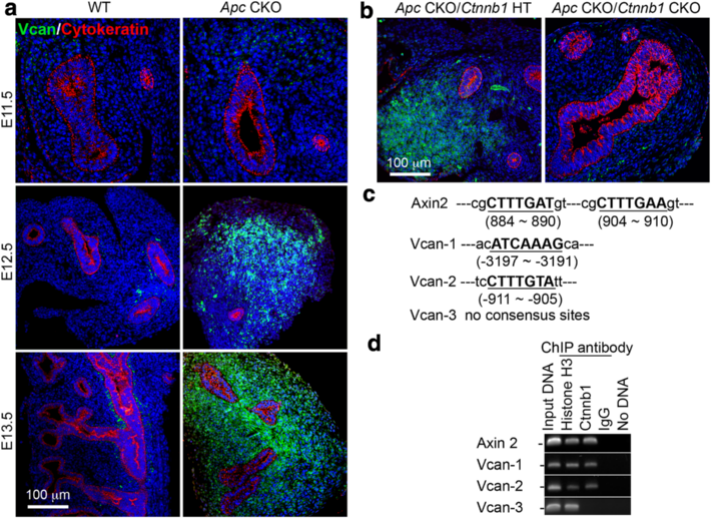
Hyperactivation of Wnt/Ctnnb1 pathway caused by *Apc* deletion in lung mesenchyme induced abnormal expression of Vcan. **a** Increased Vcan protein expression and distribution were detected in *Apc* CKO lung after E12.5, shown by immunofluorescence staining. Cell nuclei were counterstained with DAPI (blue). **b** Simultaneous deletion of *Ctnnb1* in E13.5 *Apc* CKO lung mesenchyme blocked excessive *Vcan* expression. **c** Two sites containing Ctnnb1 binding DNA consensus sequences, which have been reported in Ctnnb1 target gene *Axin2*, were identified in *Vcan* promoter DNA. **d** Interaction between Ctnnb1 and *Vcan* promoter DNA was validated by ChIP using anti-active Ctnnb1 antibody. Anti-histone H3 and normal IgG were used as positive and negative controls, respectively

In addition to cellular changes, some extracellular matrix protein production and deposition were also altered in *Apc* CKO embryos after E12.5. One of these was versican (Vcan), a large chondroitin sulfate proteoglycan. Expression of *Vcan* in normal embryonic lung is restricted to a thin mesenchymal cell layer that surrounds large airways after E12.5 (Fig. 8a). However, abrogation of *Apc* in embryonic lung mesenchyme induced ubiquitous and high expression of *Vcan*, which overlapped with the pattern of excessive Ctnnb1 activation. Moreover, simultaneous abrogation of *Ctnnb1* in *Apc* CKO lung mesenchyme blocked the abnormal expression of *Vcan* (Fig. 8b), suggesting that hyperactivation of the Wnt/Ctnnb1 pathway in *Apc* CKO lung mesenchyme is responsible for this phenotypic change. In order to determine the related molecular mechanism, the *Vcan* promoter DNA sequence was analyzed. At least two Ctnnb1/TCF consensus binding sites (5′-CTTTGAT-3′ or 5′-ATCAAAG) were identified in the *Vcan* promoter (−3197 to −3191 and −911 to −905, Fig. 8c). To further confirm the direct binding of Ctnnb1/TCF to the *Vcan* promoter, chromatin immunoprecipitation (ChIP) using anti-Ctnnb1 antibody was performed for E18.5 normal lung tissue. As shown in Fig. 8d, both Ctnnb1/TCF consensus binding sites mentioned above have been shown to interact with Ctnnb1/TCF specifically, indicating that excessive expression of *Vcan* is mediated by hyperactivation of the Wnt/Ctnnb1 pathway due to loss of Apc inhibitory function.

### Abrogation of mesenchymal Apc function inhibited epithelial branching morphogenesis by a paracrine mechanism

**Fig. 9:**
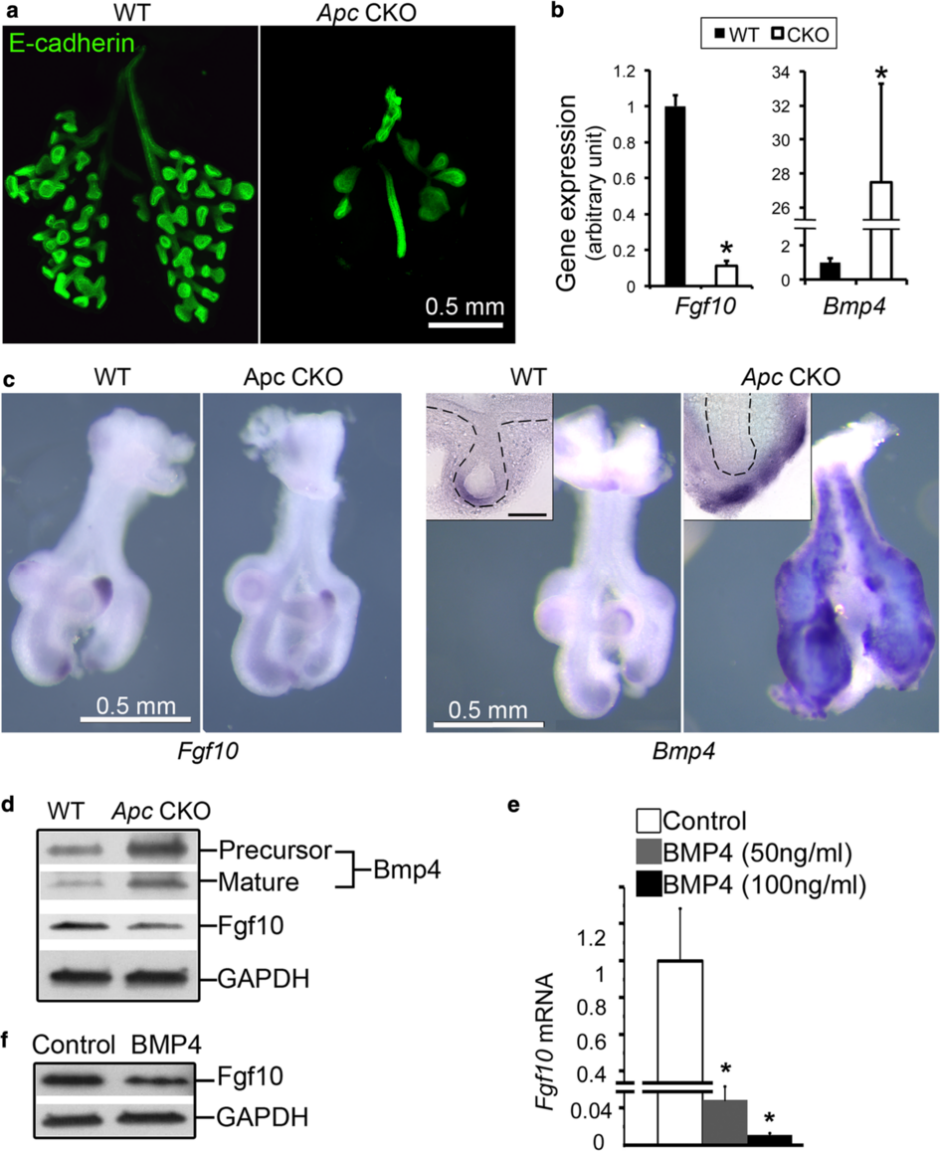
Lung epithelial branching morphogenesis was severely impaired in *Apc* CKO embryos. **a** Lung epithelial branches at E13.5 were visualized by whole mount E-cadherin immunofluorescence staining. **b** Altered expression of *Fgf10* and *Bmp4* in *Apc* CKO lung at E11.5 was detected by real-time PCR. *P < 0.05, *n* = 5. **c** Whole mount in situ hybridization showed that *Fgf10* transcript was downregulated in the mesenchymal tips of *Apc* CKO lung at E11.5. In contrast, *Bmp4* expression was dramatically increased in the sub-mesothelial mesenchyme of *Apc* CKO lung. Insert: distal tip in the vibratome section. Scale bar: 100 μm. **d** Alterations of *Fgf10* and *Bmp4* expression in E13.5 *Apc* CKO lung were verified at the protein level by western blot. **e-f** Treatment of cultured human fetal lung fibroblasts HLF1 with BMP4 (50–100 ng/ml) inhibited *Fgf10* expression at the mRNA level (**e**, measured by real-time PCR) and the protein level (**f**, detected by western blot). *P < 0.05, *n* = 5

Lung epithelial branching morphogenesis requires coordinated signaling between epithelial and mesenchymal cells. Mesenchymal *Apc* CKO resulted in smaller lung size at E13.5 (Fig. 2). The change in airway epithelial structure was visualized by whole mount E-cadherin immunofluorescence staining (Fig. 9a). Compared to WT lungs, elongation of the primary epithelial tubes and terminal bud sprouting were drastically inhibited in the *Apc* CKO lungs. In order to understand the molecular mechanisms by which altered mesenchymal Apc/Ctnnb1 activity disrupts epithelial branching, expression of genes encoding key epithelial growth factors was examined. A substantial decrease of *Fgf10* expression (>5 fold) and a striking increase of *Bmp4* expression (>20 fold) at the mRNA level were detected in *Apc* CKO lungs as early as E11.5, before morphological changes had occurred (Fig. 9b). Furthermore, whole-mount in situ hybridization showed that *Fgf10* expression domains at the distal tips of the lung bud mesenchyme were reduced or even absent (Fig. 9c), compared to those in the WT controls. In contrast, pronounced *Bmp4* expression was detected throughout the entire lung mesenchyme at E11.5, compared to the relatively restricted pattern of *Bmp4* expression to the tips of epithelial cells in WT lung (Fig. 9c). Changes of Bmp4 and Fgf10 protein expression in the entire lung tissue at E13.5 were also confirmed by western blot (Fig. 9d). Since *Bmp4* expression can be directly upregulated by Wnt/Ctnnb1 signaling in embryonic lung mesenchyme [16], we then wondered whether defective *Fgf10* expression in the *Apc* CKO lung could be mediated by increased *Bmp4*. Therefore, the regulatory effect of Bmp4 on *Fgf10* gene expression in cultured human fetal lung fibroblast line HLF1 was examined. High concentrations of BMP4 (50–100 ng/ml) were able to inhibit *Fgf10* expression at both mRNA and protein levels (Fig. 9e,f).

### Loss of mesenchymal Apc function also disrupts lung vasculogenesis and formation of the pulmonary circulation by a paracrine mechanism

**Fig. 10:**
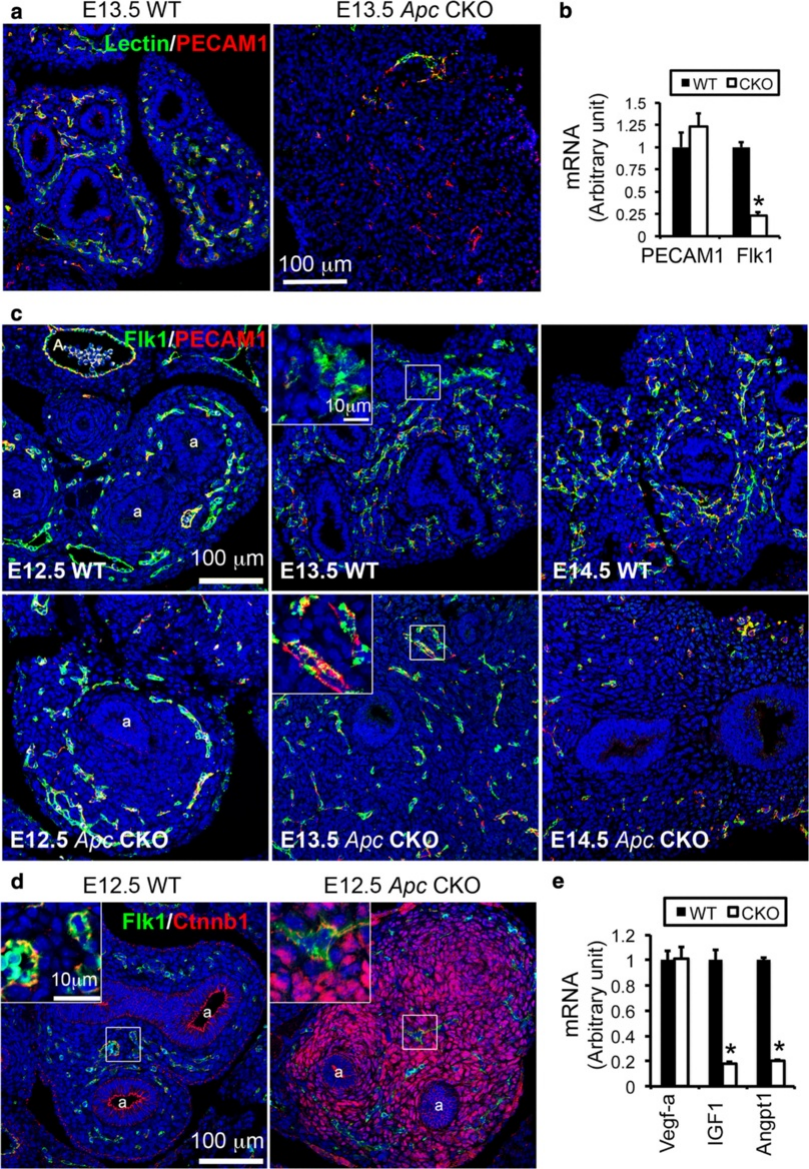
*Apc* knockout in lung mesenchyme disrupted pulmonary vascular network formation and pulmonary circulation continuity. **a** The endothelial cells of E13.5 pulmonary vasculature that was connected to the right heart were labeled with FITC-lectin after its intracardial injection, while all mature vascular endothelial cells were immunostained with anti-PECAM1. **b** Decreased expression of *Flk1* at the mRNA level was readily detected in E12.5 *Apc* CKO lungs, *P < 0.05, *n* = 5. **c** Dynamic changes of angioblasts (Flk1^+^/PECAM1^−^) and mature endothelial cells (Flk1^+^/PECAM1^+^) in *Apc* CKO lung. Reduced angioblasts were detected in *Apc* CKO lung at E13.5, while both angioblasts and mature endothelial cell numbers appeared significantly reduced in *Apc* CKO lung at E14.5. **d** Increased Ctnnb1 activation was not detected in Flk1^+^ cells in *Apc* CKO lung. **e** Reduced expression of *Igf1* and *Angpt1* at the mRNA level was detected in *Apc* CKO lungs at E12.5 (*P < 0.05, *n* = 5)

Massive lung hemorrhage in *Apc* CKO fetuses occurred around E14.5 (Figs. 2 and 3), when the pulmonary circulation should have started. This suggests that mesenchymal Apc function is essential for regulating lung vasculogenesis and/or angiogenesis as well as for building intact pulmonary circulation networks. In order to determine the vascular continuity in *Apc* CKO lungs, fluorescein isothiocyanate (FITC)-labeled lectin, an endothelial tracer, was injected into the right cardiac ventricle of E13.5 live fetuses 5 min before lung harvest, and pulmonary vascular perfusion was visualized by FITC-lectin binding. In addition, all the vasculature within the same lung tissue section was labeled by PECAM1 staining (Fig. 10a). We found that the proximal large vessels in *Apc* CKO lungs were readily perfused (Lectin^+^/PECAM1^+^), while the distal small vessels were not (Lectin^−^/PECAM1^+^). In contrast, a plexus-like pattern of lectin-labeled vasculature perfectly matched the PECAM1-stained endothelial cells in the WT lungs, indicating thorough perfusion and a well-established pulmonary circulation at this stage. Therefore, lung mesenchymal *Apc* CKO fetuses had disrupted pulmonary circulation due to disconnection between proximal vessels and the distal vasculature, which could result in the massive pulmonary hemorrhage and loss of blood, and eventually fetal lethality.

To further understand these related mechanisms, we then investigated the dynamic changes of lung vascular formation from E12.5 to E14.5 in *Apc* CKO mice by detecting two different endothelial cells markers: Flk1 and PECAM1. Flk1 is present in both pre-mature and mature endothelial cells, while PECAM1 is only expressed in mature endothelial cells [17]. Thus, Flk1^+^/PECAM1^−^ cells represent endothelial progenitors (angioblasts) and Flk1^+^/PECAM1^+^ cells are mature endothelial cells. At E12.5, although both WT and *Apc* CKO lungs had comparable patterns of Flk1 and PECAM1 protein immunostaining, with the majority of cells being Flk1^+^/PECAM1^+^, Flk1 expression at the mRNA level in *Apc* CKO lung was already reduced significantly (Fig. 10b,c). One day later (E13.5), Flk1^+^/PECAM1^−^ cells in *Apc* CKO lung were decreased, accompanied by an overall reduction and simplification of the peripheral vascular network (Fig. 10c). Furthermore, by E14.5, a marked reduction of the entire vascular network was seen in *Apc* CKO lung, including reductions in both Flk1^+^/PECAM1^+^ and Flk1^+^/PECAM1^−^ cells.

Studies have shown that the pulmonary circulation network may be formed by two coordinated mechanisms, proximal angiogenesis and distal vasculogenesis [2]. Our recent study using the Tbx4-rtTA*/*TetO-Cre*/*mT-mG reporter mice with Dox induction from E6.5 suggests that early lung mesenchymal progenitor cells give rise to the endothelial progenitor cells for vascular formation [11]. However, using the same driver/reporter line, we found that Dox induction from E10.5 did not mark both premature and mature endothelial cells with green fluorescent protein (GFP) (Additional file 7), and therefore, deletion of the *Apc* gene in our *Apc* CKO mice should not occur in these endothelial cells. This was further verified by no change of Ctnnb1 activation in Flk1^+^ cells of E12.5 *Apc* CKO lungs induced from E10.5 (Fig. 10d). Thus, the effect of mesenchymal *Apc* conditional knockout on vascular development was indirect, possibly through a paracrine mechanism. We then screened for changes of key growth factors that are important in vasculogenesis. Surprisingly, there was no difference in *Vegf-a* expression between WT and *Apc* CKO lungs (Fig. 10e). However, expression of *Igf1* and *Angpt1* at the mRNA level was markedly reduced in E12.5 *Apc* CKO lungs, suggesting that deregulation of multiple growth factors may disrupt pulmonary vascular formation in this model system.

## Discussion

Although extensive studies have been performed to determine Apc functions in epithelial progenitor/stem cell biology and tumorigenesis, the roles of Apc in mesenchymal cells during development and tissue homeostasis have not been fully investigated. It is known that loss of Apc function in mesenchymal progenitor cells in adults causes desmoid tumors, as seen in patients with familial adenomatous polyposis. However, the role of mesenchymal Apc in regulating tissue development and organogenesis has not been fully investigated. Immunohistochemical results of a previous study indicated that expression of *Apc* was restricted to embryonic lung epithelial cells [10], raising the question of whether lung mesenchymal Apc is necessary during early lung branching morphogenesis. However, by generating lung mesenchyme-specific genetic deletion of *Apc* during development, our current study suggests that *Apc* expression in early embryonic lung mesenchymal cells is in fact essential for both lung branching morphogenesis and pulmonary circulation establishment through a variety of mechanisms. Thus, previous failure to detect Apc protein in early lung mesenchyme could have resulted from low endogenous levels of Apc and/or low affinity of the antibody used for immunostaining detection.

Apc is a major negative regulator of Wnt signaling, as it directly binds to Ctnnb1 and forms a multi-protein complex with casein kinase Iα, glycogen synthase kinase 3, and Axin, resulting in Ctnnb1 phosphorylation and ubiquitination-mediated degradation [18]. Therefore, appropriate levels of Apc can provide a fine-tuning for Wnt/Ctnnb1 activity. In general, hyperactivation of Wnt/Ctnnb1 promotes cell cycle progression, resulting in increased cell proliferation and decreased cell differentiation [19]. For example, ectopic expression of constitutively active Ctnnb1 in fetal lung epithelial cells results in highly proliferative cuboidal epithelia that lack fully differentiated lung cell types [9], while blockade of canonical Wnt signaling in lung mesenchymal cells by deleting Ctnnb1 causes cell arrest at G1/S and reduced lung growth [20]. In our present study, we found that specific abrogation of *Apc* in lung mesenchymal cells caused an immediate increase in cell proliferation at E11.5 (Fig. 5), leading to the massive expansion of mesenchymal cells observed in E12.5 lung (Fig. 3). This might be attributed to activation of Wnt/Ctnnb1 signaling, as shown by accumulation of non-phospho (active) Ctnnb1 and concomitantly increased expression of its target gene *c-Myc* (Fig. 6 and Additional file 5), a cell-cycle effector that contributes to G1 progression. However, such increased cell proliferation was not sustained, and instead, cell proliferation was then reduced by E12.5 and lung growth was then arrested (Figs. 5 and 6). Interestingly, gain-of-function mutation in *Ctnnb1* by deleting its exon 3 in lung mesenchymal cells resulted in a prolonged increase in cell proliferation, with no subsequent decrease of cell proliferation, as observed as late as E13.5 (Fig. 5 and Additional file 4). Thus, one possibility is that cell growth arrest in the *Apc* CKO lung is Wnt/Ctnnb1 independent. Alternatively, deletion of the microtubule binding domain in our *Apc* mutant might suggest another potential mechanism for reduced cell proliferation, since this domain mediates nuclear spindle assembly and chromatin segregation during mitosis [21]. However, cells in metaphase were not increased in our *Apc* CKO lungs; instead, most of the *Apc* CKO mesenchymal cells were arrested in G0/G1 phase at E13.5 (Fig. 6). Furthermore, expression of both *Ccnd1* and *c-Myc* genes in our *Apc* CKO lungs was significantly reduced after E12.5 or later by an unknown mechanism. Thus, considering all these results, deletion of *Apc* in embryonic lung mesenchyme appears to directly promote cell proliferation by activating Wnt/Ctnnb1 signaling, followed by indirect inhibition of the cell cycle at G0/G1 by downregulation of *Ccnd1* and *c-Myc*.

In addition to affecting cell proliferation, *Apc* knockout also disrupted lung mesenchymal cell differentiation, in particular, airway and vascular smooth muscle cell formation. Interestingly, Carraro et al. reported that increased *Apc* expression accompanied by downregulation of Wnt/Ctnnb1 activity, achieved by knocking down miR-142-3p, promoted parabronchial smooth muscle cell progenitor differentiation [22]. Therefore, it seems that Apc functions as a positive regulator for airway smooth muscle cell differentiation by inhibiting Wnt/Ctnnb1 activity. Sox9-positive progenitor cells in normal peripheral lung are restricted to distal epithelial cells. Interestingly, *Apc* knockout resulted in Sox9-positive mesenchymal cells disseminated in distal lung mesenchyme, which were negative for another chondrocyte marker collagen II (data not included). The identity of those Sox9-positive mesenchymal cells in peripheral lung remains to be determined. It has been reported that Wnt/Ctnnb1 does not regulate Sox9 expression in lung epithelial cells, unlike in intestinal epithelial cells [23]. Considering the different patterns of Ctnnb1 activation in all cells versus sparse Sox9-positive cells in *Apc* CKO lung mesenchyme (Figs. 1 and 7), expression of Sox9 may not be due to Wnt/Ctnnb1 activation in lung mesenchymal cells. In addition, loss of Apc function also causes excessive expression of *Vcan*, a proteoglycan core molecule that has been shown to play a key role in regulating tissue development [24]. Upregulated *Vcan* expression is related to Wnt/Ctnnb1-stimulated fibroblast aggregative growth *in vitro* [25], and to inhibition of neural crest migration through its anti-adhesive activity [26]. In our study, lung mesenchymal *Apc* CKO led to aberrant activation of Ctnnb1 and *Vcan* expression, which may result in aggregative growth of lung mesenchyme and inhibition of epithelial cell migration on the distal tips and branching *in vivo*. For the first time, we also mapped Ctnnb1/TCF binding sites on a *Vcan* promoter. Taken together, our data suggest that Apc functions as a key factor to control lung mesenchymal cell lineage commitment and differentiation possibly through both Wnt/Ctnnb1-dependent and Wnt/Ctnnb1-independent pathways.

In addition to affecting mesenchymal cell biology, abrogation of mesenchymal Apc function also inhibited epithelial growth by disrupting multiple evolutionarily conserved growth factors, including Fgf and Bmps. It has been reported that mesenchymal Fgf10 directly promotes both proliferation and chemotaxis of epithelial cells on the branching tips, accompanied by indirect induction of epithelial *Bmp4* expression. As a result, mesenchymal *Fgf10* expression at the tip is then inhibited through unknown mechanisms [27]. Now for the first time, we have shown that a high level of BMP4 was able to inhibit *Fgf10* expression in cultured fetal lung fibroblasts, which is also consistent with our observation of opposing changes in *Bmp4* and *Fgf10* gene expression detected in our *Apc* CKO lungs (Fig. 9).

In parallel with airway development, lung vasculogenesis and pulmonary circulation establishment are the other important events necessary to form a functional lung. Studies have shown that pulmonary arteries grow into the lung from the hilum by angiogenic sprouting from the dorsal aorta or aortic sac [28], whereas in the distal lung, vasculogenesis creates a capillary bed in the foregut mesoderm [29]. The distal and proximal components are then fused at E13-E14 in mice to complete the pulmonary vascular circuit. Therefore, compromised continuity of the vasculature due to disconnection between distal and proximal vessels in the *Apc* CKO lungs likely led to vascular leakage around E14 when pulmonary circulation is initiated. This impaired continuity was also confirmed by observing trypan blue dye leakage in *Apc* CKO lungs after its intracardiac injection (data not shown). This could explain why massive hemorrhage into the thoracic cavity occurred one day later (E14.5, Figs. 2 and 3). Interestingly, it has been reported that blockade of Wnt ligand production from fetal lung epithelial cells by deleting Wntless (or Gpr177) also results in pulmonary hemorrhage, but such lesion occurs at a late developmental stage (neonatal) with less severity (interstitial capillary leakage without destruction of saccular structure) [30, 31]. It appears that pulmonary hemorrhage caused by lung mesenchymal *Apc* deletion versus epithelial Wntless knockout is mediated by different mechanisms. Vascular development is a complicated and multistep process that includes commitment of primitive progenitor cells to vascular progenitors [29]. By detecting dynamic changes in both angioblasts and mature endothelial cells, we found that the *Apc* CKO lung has an initially reduced population of vascular progenitors, and eventually a deficiency of mature endothelial cells (Fig. 10). These changes are not directly caused by *Apc* deletion in the endothelial linage because the Tbx4-rtTA driver line is not able to target the endothelial cell lineage after E10.5 (Additional file 7). Instead, a paracrine mechanism originating in affected lung mesenchymal cells must to be involved in this phenotypic generation. Igf1 is a potently angiogenic peptide to fetal lung endothelial cells [32], which was reduced in our *Apc* CKO lungs (Fig. 10). In addition, reduced *Angpt1* expression in *Apc* CKO lung could be another mechanism, since defects of *Angpt1* or its receptor *Tie2* result in impairment of vascular network remodeling and embryonic death [33]. Angpt1 is also related to resistance to vascular leakage [34]. Therefore, reduced expression of *Igf1* and *Angpt1* may be an important molecular mechanism underlying defective vasculogenesis and disconnected pulmonary circulation in the *Apc* CKO lungs.

## Conclusion

**Fig. 11:**
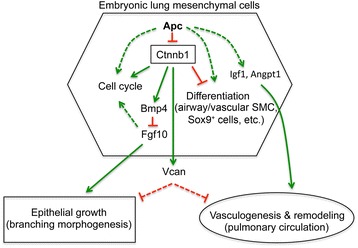
The postulated mechanisms of mesenchymal Apc as a central player in early lung development. Mesenchymal Apc not only regulates mesenchymal cell biology but also coordinates development of other cell compartments through both Wnt/Ctnnb1-dependent and Wnt/Ctnnb1-independent pathways

Apc in early embryonic lung mesenchymal cells plays a critical and diverse role in regulating lung epithelial branching morphogenesis and mesenchymal growth as well as pulmonary vascular network formation through both Wnt/Ctnnb1-dependent and Wnt/Ctnnb1-independent mechanisms (Fig. 11).

## Methods

### Mouse strains, breeding, and genotyping

Lung mesenchyme-specific *Apc* CKO mice were generated at the animal facility of Children’s Hospital Los Angeles by crossing homozygous *Apc*^*fx/fx*^ mice and the triple transgenic driver line (*Apc*^*fx/wt*^*/*Tbx4-rtTA/TetO-Cre), with Dox induction (625 mg/kg in food (TestDiet) and 0.5 mg/ml in drinking water (Sigma)) from E10.5. *Floxed-Apc* (*Apc*^fx/fx^) mice and *mT-mG* double fluorescence Cre reporter mice were obtained from Jackson Laboratory [7, 35]. *Tbx4-rtTA* was generated in our lab [11]. The TetO-Cre mouse line was provided by Dr. Jeffrey Whitsett at Cincinnati Children’s Hospital [36]. Mice with one of the following tail DNA genotypes: *Apc*^*fx/fx*^, *Apc*^*fx/fx*^*/Tbx4-rtTA*, *Apc*^*fx/fx*^*/TetO-Cre*, *Apc*^*fx/wt*^, *Apc*^*fx/wt*^*/Tbx4-rtTA,* or *APC*^*fx/wt*^*/TetO-Cre*, had no deletion of *Apc* allele and displayed normal lung morphogenesis, and therefore were grouped as the “WT” control. Cre-mediated deletion of *Apc* exon 14 leads to premature termination of Apc peptide, and most functional domains including binding sites for Ctnnb1 and microtubule were truncated [7]. For generation of *Apc*/*Ctnnb1* double knockout in lung mesenchyme, double heterozygous (*Apc*^*fx/wt*^/*Ctnnb1*^*fx/wt*^) mice carrying Tbx4-rtTA/TetO-Cre transgenes were crossed with each other, with Dox induction from E10.5. The *Ctnnb1*^*fx/fx*^ mice, with floxed exon 2 to exon 6, were obtained from Jackson Laboratory [37].

Mice with lung mesenchyme-specific constitutively active Ctnnb1 expression were generated at Justus Liebig University Giessen, by crossing *Tbx4-Cre*^ERT2^ transgenic mice and the mice with floxed exon 3 of *Ctnnb1* (*Ctnnb*^fx-ex3^). Deletion of *Ctnnb1* exon 3 was achieved by a single intraperitoneal (IP) injection of tamoxifen (100 mg/kg body weight, Sigma) at E10.5, which resulted in expression of a constitutively active form of Ctnnb1 [38]. *Tbx4-Cre*^ERT2^ mice, with the same *Tbx4* lung enhancer as Tbx4-rtTA above, were provided by Dr. Mark Krasnow at Stanford University, and *Ctnnb*^fx-ex3^ mice were obtained from Dr. Mark Taketo [38].

All mice were bred in C57BL/6 strain background. The mice used in this study were housed in pathogen-free facilities. All procedures were approved by the related Institutional Animal Care and Use Committees.

### Histology and immunofluorescence analysis

Morphological analysis was conducted as previously described [11]. Immunofluorescence staining was performed following the methods published previously [39]. The related antibodies are listed in Additional file 8. Whole mount E-cadherin immunostaining was used to visualize lung epithelial branching following a modified protocol published previously [40]. Fluorescence images were taken using the Zeiss LSM710 confocal microscope at the Imaging Core Facility of Children’s Hospital Los Angeles. Flk1^+^/PECAM1^−^, Flk1^+^/PECAM1^+^, and DAPI^+^ cells were counted under 200X magnification using a Cell Counter plugin of Fiji imaging software (1.48q). Cell counting was repeated with four mice in each genotype group.

### Cell proliferation, apoptosis, and DNA content analyses

For *in vivo* cell proliferation assay, pregnant mice were intraperitoneally (IP) injected with 5′-ethynyl-2′-deoxyuridine (EdU, 5 mg/kg body weight, Life Technologies), which is thymidine analogue incorporated into newly synthesized DNA. For short-term labeling, EdU was administrated 2 hours before harvesting lung specimens. Incorporated EdU was detected using Alexa Fluor azide (Life Technologies), and cell nuclei were counterstained with DAPI. The image analysis was performed in four random fields per slide from a total of four slides per mouse by an experienced observer blind to the mouse genotype. The ratios of EdU-labeled nuclei to total nuclei (n > 300 per field for mesenchymal cells and n > 50 per field for epithelial cells) were used to evaluate cell proliferation, and the experiments were repeated in more than three mice within each genotype group. For pulse-chase experiments, IP injection of EdU was performed at E11.5, and embryonic mouse lung specimens were harvested at E13.5. The sizes of cell nuclei (n > 600 from at least three mouse lungs in each genotype group) were evaluated by quantifying their average areas under 630X magnification using Fiji imaging software (1.48q). Apoptosis was evaluated using an ApopTag kit (Trevigen). For analysis of cellular DNA content, cells from E13.5 lungs were prepared by tissue digestion, and cellular DNAs were stained with 50 μg/ml of propidium iodide (PI). Differences in DNA content were determined based on fluorescence intensity of PI by FACS. Cells that are in the G0/G1 phase have a defined amount (1×) of DNA (that is, a diploid chromosomal DNA), while cells in the G2 and M phases (G2/M) have a 2× amount of DNA (a tetraploid chromosomal DNA). Cells containing DNA between 1× and 2× amounts are in S phase.

### Cell culture, real-time PCR, and western blot

Human fetal lung fibroblast cell line (HLF1) was purchased from ATCC. The cultured cells were treated with BMP4 (50 ng/ml to 100 ng/ml final concentration, R&D Systems) overnight and then harvested for RNA and protein analyses.

Total RNA was isolated from cultured cells or lung tissues using the RNeasy Kit (Qiagen). cDNA synthesis and real-time PCR were performed as described in a previous publication [36]. The related oligonucleotide primers are listed in Additional file 9. Protein lysate was prepared from lung tissues (n = 3 per group) or cultured cells (n = 4 per group) and analyzed by western blot as previously described [41]. The related antibodies used for western blot are listed in Additional file 8.

### Whole mount in situ hybridization

Lungs were isolated from embryos and fixed for 2 hours in 4 % paraformaldehyde in phosphate buffered saline (PBS). The samples were washed twice in PBS for 5 min, transferred to 70 % ethanol overnight, and stored in 100 % ethanol until needed. Whole mount in situ hybridization was conducted as previously described, with *Fgf10* or *Bmp4* antisense riboprobes transcribed from murine *Fgf10* or *Bmp4* cDNA templates [42].

### Chromatin immunoprecipitation (ChIP)

Three E18.5 WT or *Apc* CKO lung tissues were pooled, cross-linked with 1.5 % formaldehyde, and then quenched using glycine. Chromatin DNAs were then co-precipitated with the interacted proteins of interest with antibodies against Histone H3, or active Ctnnb1 (Cell Signaling Technology). Normal rabbit IgG was used as negative control. The co-immunoprecipitated DNAs were released and purified using a SimpleChIP® Plus Enzymatic Chromatin IP Kit (Cell Signaling Technology) and analyzed by PCR with the primers listed in Additional file 10. The experiments were repeated three times.

### In utero cardiac injection of mouse embryos

Pulmonary vascular perfusion followed the method described by Shah [43]. In brief, the pregnant mouse was anesthetized and subjected to a laparotomy, in which the uterus was exposed and a single embryo was isolated. The fetal mice were subjected to thoracotomy. 5.0 μl of FITC-labeled lectin (100 μg/ml, Vector Laboratories) in saline containing 1 mg/ml trypan blue was injected to the right ventricle using a fine glass needle. The injected lectin was allowed to circulate for 5 min for its binding to the endothelial wall of the vasculature. Then, the embryos were dissected, fixed with 4 % buffered paraformaldehyde, and embedded in OCT for tissue frozen section.

### Statistical analysis

All quantitative data were presented as mean ± s.d. Statistical analyses were performed using Student’s *t*-tests, with *P* ≤ 0.05 considered significant.

## Additional files

Additional file 1:
**Accumulation of active Ctnnb1 in E12.5 lung mesenchymal cells was verified using an alternative anti-nonphospho-Ctnnb1 (Ser37/Thr41) antibody from Millipore, which was different from the anti-nonphospho-Ctnnb1 (Ser33/37/Thr41) antibody (Cell Signaling Technology) used in Fig.** 1d **.**
Additional file 2:
**Altered lung mesenchymal cell density and orientation in lung mesenchyme-specific** ***Apc*** **CKO embryos at E12.5 and E13.5.**
Additional file 3:
**Changes in E14.5** ***Apc*** **CKO mouse lung, shown by a series of H&E-stained horizontal tissue sections.**
Additional file 4:
**Cell proliferation was compared between E11.5 WT lungs and mesenchyme-specific constitutively active** ***Ctnnb1*** **lungs by 2-hour EdU labeling.** Epithelial tubes were highlighted with dashed lines. Additional file 5:
**Alterations of cellular primary cilia (A) and c-Myc expression (B) in** ***Apc*** **CKO lung from E11.5 to E12.5 were shown by immunofluorescence staining using the indicated antibodies.** Cell nuclei were counterstained with DAPI (blue). Additional file 6:
**No change in apoptosis was seen in E13.5** ***Apc*** **CKO lung compared to WT controls, shown by TUNEL.**
Additional file 7:
**The Tbx4-rtTA-driven Tet-On system with Dox induction after E10.5 was not able to directly target endothelial cells.** Tbx4-rtTA targeted cells expressed mGFP (green) in the E11.5 Tbx4-rtTA/TetO-Cre/mT-mG reporter mouse lung with Dox induction from E10.5, while endothelial cells were detected by PECAM1 or Flk1 immunostaining (red). Overlap between GFP and endothelial marker was not detected. Additional file 8:
**Primary antibodies used for immunochemistry and western blot.**
Additional file 9:
**DNA primers used for real-time RT-PCR.**
Additional file 10:
**DNA primers used in ChIP assay.**

## Abbreviations

Angpt1: angiopoietin 1
*Apc*: *adenomatous polyposis coli*
BMP: bone morphogenetic protein
ChIP: chromatin co-immunoprecipitation
CKO: conditional knockout
DAPI: 4′,6-diamidino-2-phenylindole
Dox: doxycycline
E: embryonic day
EdU: 5-ethynyl-2-deoxyuridine
FGF: fibroblast growth factor
FITC: fluorescein isothiocyanate
Igf1: insulin-like growth factor 1
mT-mG: *loxP*-mTomato-STOP-*loxP*-mGFP
PECAM1: platelet endothelial cell adhesion molecule 1
rtTA: reverse tetracycline transactivator
SMA: α-smooth muscle actin
Vcan: versican
Vegf-a: vascular endothelial growth factor A
WT: wild type
