# Supplementary material for: Mesenchymal adenomatous polyposis coli plays critical and diverse roles in regulating lung development
Source: BMC Biol. 2015 Jun 20;13:42. doi: 10.1186/s12915-015-0153-1 (PMC4702410; doi:10.1186/s12915-015-0153-1)
Supplement: Additional file 2: — Altered lung mesenchymal cell density and orientation in lung mesenchyme-specific Apc CKO embryos at E12.5 and E13.5. [file 12915_2015_153_MOESM2_ESM.docx]

**
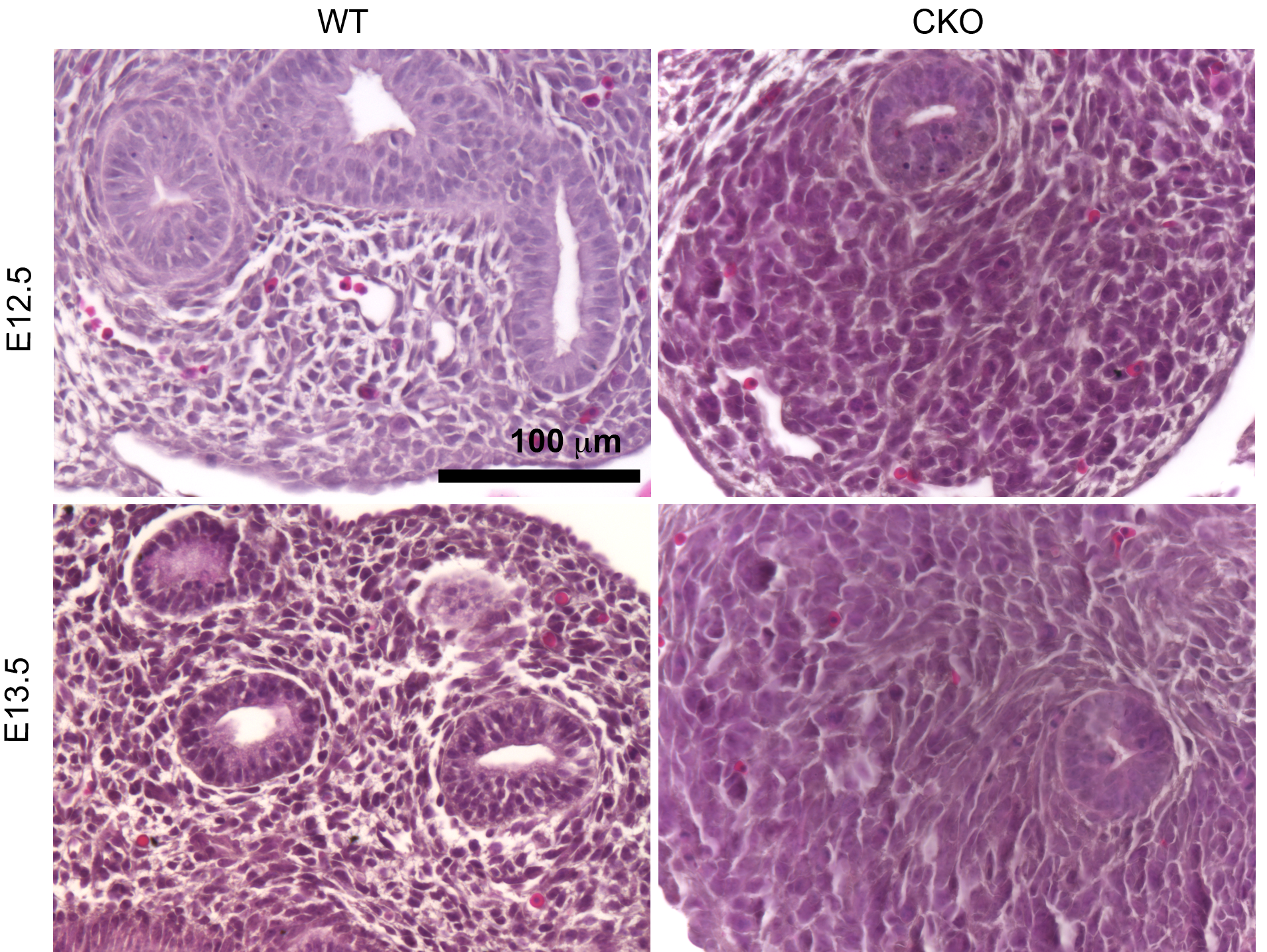
**

**Additional file 2:** Altered lung mesenchymal cell density and orientation in lung mesenchyme-specific *Apc* CKO embryos at E12.5 and E13.5.
