# Supplementary material for: Mesenchymal adenomatous polyposis coli plays critical and diverse roles in regulating lung development
Source: BMC Biol. 2015 Jun 20;13:42. doi: 10.1186/s12915-015-0153-1 (PMC4702410; doi:10.1186/s12915-015-0153-1)
Supplement: Additional file 3: — Changes in E14.5 Apc CKO mouse lung, shown by a series of H&E-stained horizontal tissue sections. [file 12915_2015_153_MOESM3_ESM.docx]

**
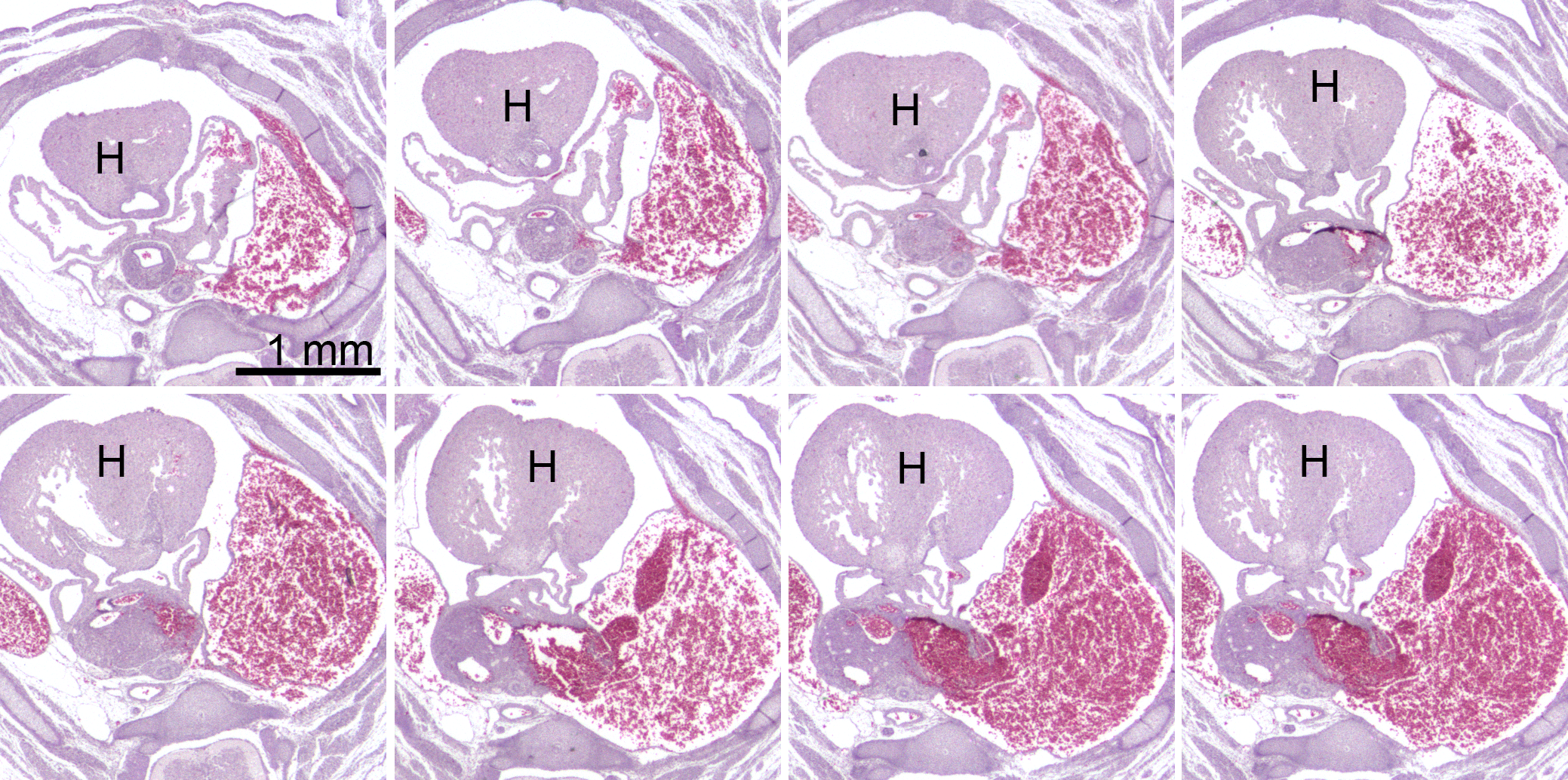
**

**Additional file 3:** Changes in E14.5 *Apc* CKO mouse lung, shown by a series of H&E-stained horizontal tissue sections.
