# Supplementary material for: Mesenchymal adenomatous polyposis coli plays critical and diverse roles in regulating lung development
Source: BMC Biol. 2015 Jun 20;13:42. doi: 10.1186/s12915-015-0153-1 (PMC4702410; doi:10.1186/s12915-015-0153-1)
Supplement: Additional file 4: — Cell proliferation was compared between E11.5 WT lungs and mesenchyme-specific constitutively active Ctnnb1 lungs by 2-hour EdU labeling. Epithelial tubes were highlighted with dashed lines. [file 12915_2015_153_MOESM4_ESM.docx]

**
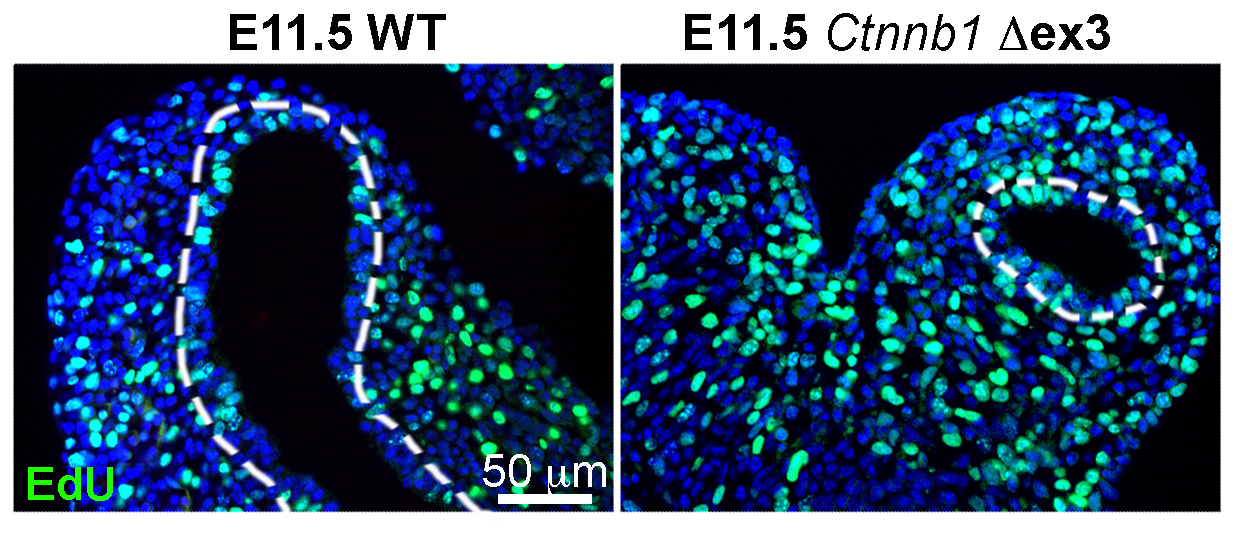
**

**Additional file 4:** Cell proliferation was compared between E11.5 WT lungs and mesenchyme-specific constitutively active *Ctnnb1* lungs by 2-hour EdU labeling. Epithelial tubes were highlighted with dot lines.
