# Supplementary material for: Mesenchymal adenomatous polyposis coli plays critical and diverse roles in regulating lung development
Source: BMC Biol. 2015 Jun 20;13:42. doi: 10.1186/s12915-015-0153-1 (PMC4702410; doi:10.1186/s12915-015-0153-1)
Supplement: Additional file 5: — Alterations of cellular primary cilia (A) and c-Myc expression (B) in Apc CKO lung from E11.5 to E12.5 were shown by immunofluorescence staining using the indicated antibodies. Cell nuclei were counterstained with DAPI (blue). [file 12915_2015_153_MOESM5_ESM.docx]

**
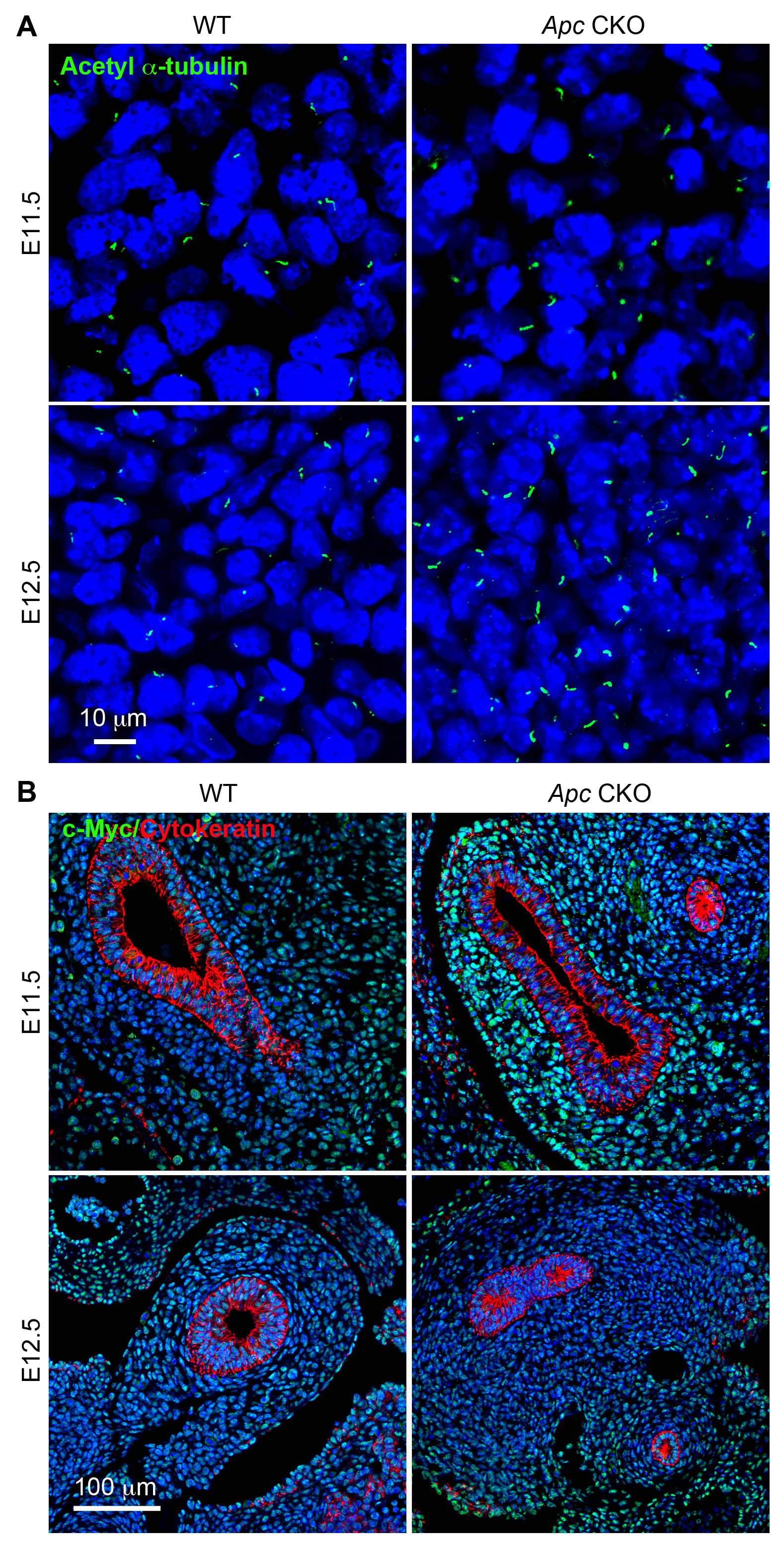
**

**Additional file 5:** Alterations of cellular primary cilia (A) and c-Myc expression (B) in *Apc* CKO lung from E11.5 to E12.5 were shown by immunofluorescence staining using the indicated antibodies. Cell nuclei were counterstained with DAPI (blue).
