# Supplementary material for: Mesenchymal adenomatous polyposis coli plays critical and diverse roles in regulating lung development
Source: BMC Biol. 2015 Jun 20;13:42. doi: 10.1186/s12915-015-0153-1 (PMC4702410; doi:10.1186/s12915-015-0153-1)
Supplement: Additional file 6: — No change in apoptosis was seen in E13.5 Apc CKO lung compared to WT controls, shown by TUNEL. [file 12915_2015_153_MOESM6_ESM.docx]

**
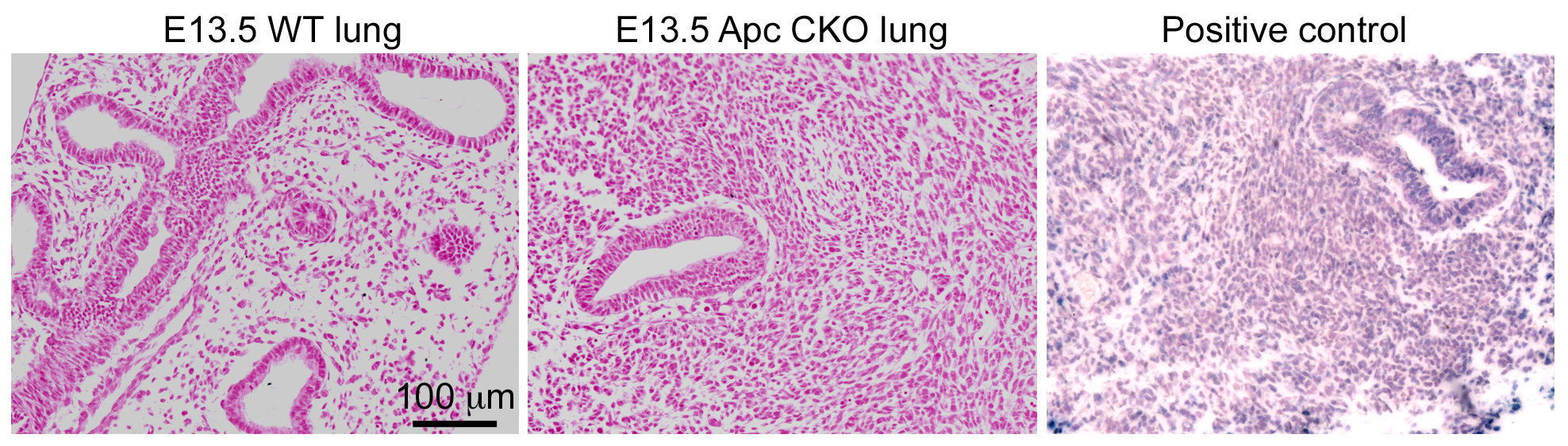
**

**Additional file 6:** No change in apoptosis was seen in E13.5 *Apc* CKO lung compared to WT controls, shown by TUNEL.
