# Supplementary material for: Mesenchymal adenomatous polyposis coli plays critical and diverse roles in regulating lung development
Source: BMC Biol. 2015 Jun 20;13:42. doi: 10.1186/s12915-015-0153-1 (PMC4702410; doi:10.1186/s12915-015-0153-1)
Supplement: Additional file 8: — Primary antibodies used for immunochemistry and western blot. [file 12915_2015_153_MOESM8_ESM.docx]

**Additional file 8:**

Primary antibodies used for immunochemistry

| Antibody name | Vendor | Catolog |
| --- | --- | --- |
| Mouse anti-EGFP | Clontech | #632569 |
| Rabbit anti-RFP | Rockland | 600-401-379 |
| Goat anti-Pecam | Santa Cruz | Sc-1506 |
| Rabbit anti-Non-phospho(Active) β-Catenin (Ser33/37/Thr41) | Cell Signaling Technology | #8814 |
| Rabbit anti-active β-Catenin | Millipore | #05-665 |
| Rabbit anti-Axin 2 | LSBio | LS-B6746 |
| Mouse anti-Cytokeratin | Sigma | C2562 |
| Rabbit anti-Phospho-Histone H3 (Ser10) | Cell Signaling Technology | #9701 |
| Mouse anti-Acetylated α-tubulin | Sigma | T7451 |
| Rabbit anti-c-Myc | LSBio | LS-B7667 |
| Mouse anti-SMA | Sigma | A2547 |
| Rabbit anti-Sox2 | Santa Cruz | sc-20088 |
| Rabbit anti-Sox9 | Santa Cruz | sc-20095 |
| Rabbit anti-versican | Millipore | AB1032 |
| Rat anti-Cadherin | Calbiochem | #205604 |
| goat anti-Flk-1 | LSBio | LS-C150271 |
| Rabbit anti-Pecam | Santa Cruz | LS-B1932 |

Primary antibodies used for western blot

| Antibody name | Vendor | Catolog |
| --- | --- | --- |
| Rabbit anti-c-Myc | Santa Cruz | Sc-40 |
| Mouse anti-Ccnd1 | Cell Signaling Technology | #2926 |
| Rabbit anti-Phospho-p53 (Ser15) | Cell Signaling Technology | #9284 |
| Rabbit anti-P53 (FL-393) | Santa Cruz | sc-6243 |
| Rabbit anti-Caspase-3 | Cell Signaling Technology | #9662 |
| Rabbit anti-Fgf10 | Millipore | ABN44 |
| Mouse anti-BMP4 | R&D Systems | MAB757 |
| Mouse anti-GAPDH | Fitzgerald | 10R-G109a |
