# Supplementary material for: Mesenchymal adenomatous polyposis coli plays critical and diverse roles in regulating lung development
Source: BMC Biol. 2015 Jun 20;13:42. doi: 10.1186/s12915-015-0153-1 (PMC4702410; doi:10.1186/s12915-015-0153-1)
Supplement: Additional file 9: — DNA primers used for real-time RT-PCR. [file 12915_2015_153_MOESM9_ESM.docx]

**Additional file 9:** DNA primers used for real-time RT-PCR

| Gene | Oligonucleotide DNA sequence |
| --- | --- |
| *Axin2* | 5’-TGG CTG GTG AAA AGA CAT AG-3’ |
|  | 5’-TGT GAG ATC CAC GGA AAC AG-3’ |
| *Myc* | 5’-CAG CTG GAG ATG ATG ACC GA-3’ |
|  | 5’-TTG ATG AAG GTC TCG TCG TC-3’ |
| *Ccnd1* | 5’-AGT GCC TAC AGC CCT GTT AC-3’ |
|  | 5’-TAG AGG TCG CAC TGA CCA TC-3’ |
| *Glb1* | 5’- GTG AAA AGC CTC TAT CCC CTG -3’ |
|  | 5’- ATC ACG GAC ACC ATT GAA GG -3’ |
| *Bmp4* | 5’- TCC ATC ACG AAG AAC ATC-3’ |
|  | 5’- TAG TCG TGT GAT GAG GTG-3’ |
| *Fgf10* | 5’- GAA GGG GAA ACT CTA TGG CTC A-3’ |
|  | 5’- TCC TCT CCT GGG AGC TCC TT-3’ |
| *PECAM1* | 5’- GAG ATG TCC AGG CCA GCT G-3’ |
|  | 5’- CTC ACT GTA CAC CGT CTC TG-3’ |
| *Flk1* | 5’-ACC CTC GTT TTC AGA GTT GG-3’ |
|  | 5’-GAA ACA GGT GAG GTA GGC AG-3’ |
| *Vegfa* | 5’-CTG GAC CCT GGC TTT ACT GC-3’ |
|  | 5’- TGA ACT TGA TCA CTT CAT GGG ACT-3’ |
| *Igf1* | 5’-TGG ATG CTC TTC AGT TCG TG-3’ |
|  | 5’-AGT ACA TCT CCA GTC TCC TCA G-3’ |
| *Angpt1* | 5’-ACC CTC GTT TTC AGA GTT GG-3’ |
|  | 5’-ACC GTG TAA GAT CAA GCT GC-3’ |
| *Gapdh* | 5’-GGT GGA GCC AAA AGG GTC AT-3’ |
|  | 5’-AGT TGT CAT ATT TCT CGT GGT TCA-3’ |
