# Supplementary material for: Mesenchymal adenomatous polyposis coli plays critical and diverse roles in regulating lung development
Source: BMC Biol. 2015 Jun 20;13:42. doi: 10.1186/s12915-015-0153-1 (PMC4702410; doi:10.1186/s12915-015-0153-1)
Supplement: Additional file 10: — DNA primers used in ChIP assay. [file 12915_2015_153_MOESM10_ESM.docx]

**Additional file 10:** DNA primers used in ChIP assay

| Gene | DNA oligonucleotide sequences |
| --- | --- |
| Vcan-1 | 5’-GGA CAC CTG CCA CCA ATA TCT-3’ |
|  | 5’- AGC CCA AGG CAC TAT GCA AT-3’ |
| Vcan-2 | 5’-CAA AGT CGC CCT TTT GTG AGG-3’ |
|  | 5’- GCA CAC CCT TCA CTG AAT CAC-3’ |
| Vcan-3  (negative control) | 5’-TTG TTT GAG ACT GCA CAG CG-3’ |
|  | 5’-ACC TCT CAG CGT TTC TTC CT-3’ |
| Axin2  (positive control) | 5’-AGC GGA TCA ATG GTG AGT GC-3’ |
|  | 5’- AAA TAG CCG GCC TGC CAA C-3’ |
