# Supplementary material for: Mesenchymal adenomatous polyposis coli plays critical and diverse roles in regulating lung development
Source: BMC Biol. 2015 Jun 20;13:42. doi: 10.1186/s12915-015-0153-1 (PMC4702410; doi:10.1186/s12915-015-0153-1)
Supplement: Supplementary file 11 [file 12915_2015_153_MOESM1_ESM.docx]

Additional file 1: Accumulation of active Ctnnb1 in E12.5 lung mesenchymal cells was verified using an alternative anti-nonphospho-Ctnnb1 (Ser37/Thr41) antibody from Millipore, which was different from the anti-nonphospho-Ctnnb1 (Ser33/37/Thr41) antibody (Cell Signaling) used in Fig.1D.
