# Supplementary material for: Mesenchymal adenomatous polyposis coli plays critical and diverse roles in regulating lung development
Source: BMC Biol. 2015 Jun 20;13:42. doi: 10.1186/s12915-015-0153-1 (PMC4702410; doi:10.1186/s12915-015-0153-1)
Supplement: Supplementary file 12 [file 12915_2015_153_MOESM7_ESM.docx]

Additional file 7: The Tbx4-rtTA driven Tet-On system with Dox induction after E10.5 was not able to directly target endothelial cells. Tbx4-rtTA targeted cells expressed mGFP (green) in the E11.5 Tbx4-rtTA/TetO-Cre/mT-mG reporter mouse lung with Dox induction from E10.5, while endothelial cells were detected by PECAM1 or Flk1 immunostaining (red). Overlap between GFP and endothelial marker was not detected.
